# Characterizing clinical toxicity in cancer combination therapies

**DOI:** 10.1093/bioinformatics/btag007

**Published:** 2026-01-14

**Authors:** Alexandra M Wong, Cecile P G Meier-Scherling, Lorin Crawford

**Affiliations:** Center for Computational Molecular Biology, Brown University, Providence, RI 02912, United States; Center for Computational Molecular Biology, Brown University, Providence, RI 02912, United States; Microsoft Research, Cambridge, MA 02142, United States

## Abstract

**Motivation:**

Predicting synergistic cancer drug combinations through computational methods offers a scalable approach to creating therapies that are more effective and less toxic. However, most algorithms focus solely on synergy without considering toxicity when selecting optimal drug combinations. In the absence of combinatorial toxicity assays, a few models use toxicity penalties to balance high synergy with lower toxicity. Still, these penalties have not been explicitly validated against known drug-drug interactions.

**Results:**

In this study, we examine whether synergy scores and toxicity metrics correlate with known adverse drug interactions. While some metrics show trends with toxicity levels, our results reveal significant limitations in using them as penalties. These findings highlight the challenges of incorporating toxicity into synergy prediction frameworks and suggest that advancing the field requires more comprehensive combination toxicity data.

**Availability and implementation:**

The code written for this project is available at https://github.com/amw14/toxicity-cancer-drug-combination.

## 1 Introduction

Cancer is a significant cause of mortality both in the United States as well as globally (Murphy *et al.* 2021), and its incidence is expected to increase over the next fifty years (Soerjomataram and Bray 2021). To address drug resistance, the cause of over 90% of cancer deaths (Bukowski *et al.* 2020, National Cancer Institute 2022), scientists have successfully developed combination therapies to combat tumor heterogeneity and narrow possible mechanisms for therapeutic escape (Al-Lazikani *et al.* 2012, Lopez and Banerji 2017). However, discovering novel combination therapies involves costly high-throughput screening assays, which become increasingly more expensive as the number of potential drug candidates grows (Sun *et al.* 2013, Mennen *et al.* 2019). To mediate this combinatorial explosion, *in silico* approaches have taken advantage of the large-scale multi-omic and cancer drug databases to learn to predict new therapeutic combinations (The Cancer Genome Atlas Research Network 2006, O’Neil *et al.* 2016, Holbeck *et al.* 2017, Zagidullin *et al.* 2019).

Many of these *in silico* methods use machine learning to predict a “synergy score” (Preuer *et al.* 2018, Julkunen *et al.* 2020, Ranasinghe *et al.* 2021, Wang *et al.* 2022, Rafiei *et al.* 2023, Abd El-Hafeez *et al.* 2024, Rafiei *et al.* 2024, Chen *et al.* 2025, Hao *et al.* 2025, Yan *et al.* 2025), which is a quantitative measure meant to optimize for drug-drug interactions (DDI) that produce a combined effect greater than the “additive” effect (i.e. the sum of their individual effects). However, there is increasing evidence that synergy scores may not be the best metric to optimize. First, additivity alone has been shown to be predictive of the efficacy of many approved cancer drug combinations (Palmer and Sorger 2017, Hwangbo *et al.* 2023). Second, cancer combination therapies can better accommodate the diverse nature of patient populations, increasing the likelihood that at least one drug will be effective for each patient, rather than relying on a synergistic effect in any single individual (Palmer and Sorger 2017). Additionally, what can be considered “synergistic” is variable. For example, the DrugComb database reports seven different synergy scoring methods (Zagidullin *et al.* 2019), reflecting that defining synergy remains an open question. Thus, optimizing for synergy scores alone may not be the best primary focus for predictive computational models.

Another significant hurdle in cancer therapeutics development is toxicity, which is the second biggest reason for clinical trial failure (Harrison 2016, Hwang *et al.* 2016, Sun *et al.* 2022). Even after a therapy is approved, intolerable side effects can contribute to the early discontinuation of a patient’s treatment schedule (Sella *et al.* 2022, Raychaudhuri *et al.* 2024, Virtanen *et al.* 2024). Toxicity plays a critical role in clinical translation, and drug synergy is thought to reduce toxicity based on the principle that higher synergism allows for lower dosages of each drug to create the same efficacy on the cancer, reducing the potential side effects from either individual drug (Al-Lazikani *et al.* 2012, Ma *et al.* 2014). While many methods cite reducing toxicity as a motivation for finding synergistic drug combinations, most computational models only focus on predicting a synergy value without regard to adverse drug effects (Preuer *et al.* 2018, Julkunen *et al.* 2020, Ranasinghe *et al.* 2021, Wang *et al.* 2022, Rafiei *et al.* 2024, Hao *et al.* 2025, Zhang *et al.* 2025). Fewer even attempt to integrate toxicity. One approach, SynToxProfiler, studied a prioritization scheme to balance synergy with toxicity and efficacy when ranking optimal combination candidates (Ianevski *et al.* 2020). Notably, some of their top-performing combinations had negative synergy scores (i.e. combinations that would have been missed when selecting by synergy scores alone). SynToxProfiler created toxicity scores through experimental measurements of drug combinations tested on healthy control cells. However, this type of combinatorial toxicity data is not often available for many of the large cancer drug combination datasets that are widely used in the field.

Absent that data, some combination prediction methods have sought to incorporate toxicity information in other ways. Chen *et al.* (2025) and Hosseini and Zhou (2023) used clinical side effects of drugs from the SIDER database as drug features in their combination prediction, but they did not explicitly attempt to minimize toxicity. These approaches also do not consider known adverse effects from the drug *combination*, as SIDER only contains adverse effects for single drugs. Other methods add toxicity penalties in their loss functions when predicting synergistic combinations, seeking to balance high synergy with low toxicity. DeepTraSynergy by Rafiei *et al.* (2023) and GraphSynergy by Yang *et al.* (2021) created novel loss functions based on the idea that greater drug target and pathway overlap leads to higher toxicity (Cheng *et al.* 2019). DeepTraSynergy also incorporated drug structure representations into its toxicity penalty because similarity of drug structures is also thought to increase the chances of adverse DDIs (Vilar *et al.* 2014). However, whether these metrics correspond to clinical toxicity remains uncharacterized.

In this study, we explore how well synergy scores correlate with clinically known combination toxicity. We ask three key questions. First, is there a relationship between synergy scores and toxicity levels of DDIs? This question is critical for understanding if current methods may be optimizing for more or less toxic combinations when prioritizing only by the synergy score. Second, can overlap of drug targets and pathways explain toxicity and synergy? This assessment seeks to confirm how robust target overlap is as a measure of toxicity. Finally, do common methods used in toxicity penalties correlate with known DDIs? This analysis investigates whether toxicity penalties are sufficient for balancing efficacy and safety in combination candidates. In our analysis, we show that while current measures may correlate with general toxicity trends, they are insufficient in capturing the complexity of clinically known adverse DDIs and that there is a need for more explicit combinatorial toxicity data.

## 2 Materials and methods

**Figure 1.**
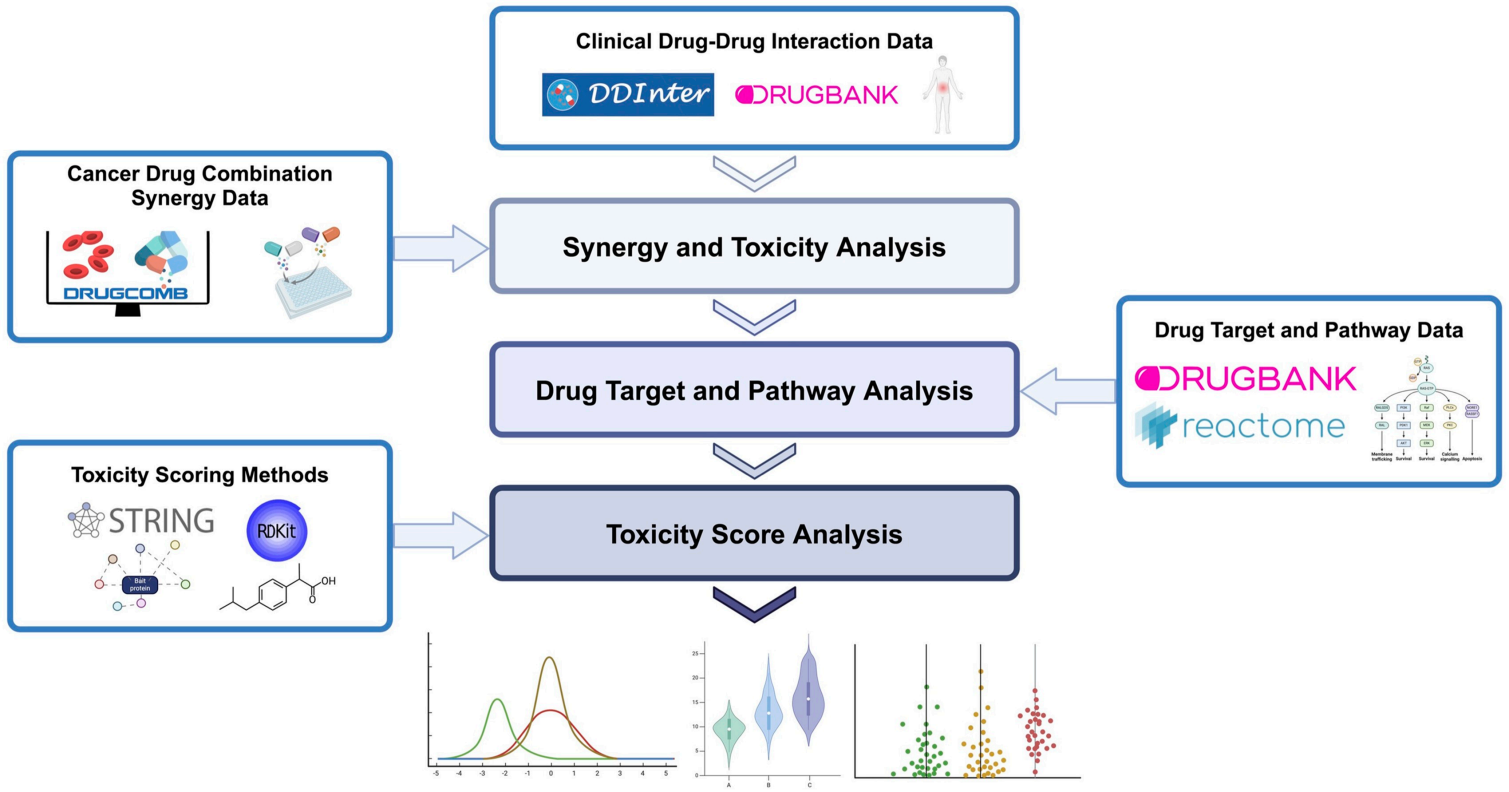
Overview of the databases and analyses conducted in this study. We first retrieved the drug-drug interactions (DDI) toxicity data from the DrugBank and DDInter databases. For the synergy and toxicity analysis, we used the toxicity data in combination with the synergy scores from the DrugComb (Zagidullin *et al.* 2019) database. For the drug target and pathway analysis, we retrieved drug target data from DrugBank and pathway information from Reactome (Milacic *et al.* 2024). For the toxicity score analysis, we used STRING (Szklarczyk *et al.* 2023) to create drug target distance metrics and acquired drug structure representations through RDKit (Landrum *et al.* 2023). Illustration created by AMW in BioRender (see https://BioRender.com/t03f351).

We used the DrugBank (Knox *et al.* 2024) and DDInter (Xiong *et al.* 2022) databases (accessed February 6th, 2025) to retrieve toxicity level information for drug combinations. We also used the DrugComb (Zagidullin *et al.* 2019) database to retrieve cancer drug combination synergy scores. Our study has three major parts: (i) an analysis of the relationship between synergy scores and toxicity, (ii) an analysis of the biological mechanisms underpinning toxicity and synergy, and (iii) an analysis of whether toxicity penalty methods correlate with known toxicity (see schematic overview in Fig. 1). Descriptions of each analysis were provided in the sections below. Details of the databases, scoring methods, and pre-processing are provided in the Supplementary Material, available as supplementary data at *Bioinformatics* online.

### 2.1 Overview of toxicity databases: DDInter and DrugBank

After filtering the toxicity databases for combinations with available synergy metrics, we retained 52 638 DrugBank and 22 457 DDInter pairs (Supplementary Methods, available as supplementary data at *Bioinformatics* online). Both datasets were integrated by standardizing drug identifiers and target annotations. Summary statistics describing synergy score distributions and network features are shown in Table S1, available as supplementary data at *Bioinformatics* online. Our initial analysis focused on DrugBank as it is the most frequently updated and comprehensive dataset. We subsequently used the DDInter dataset to validate the robustness of our core findings and to highlight important differences in how toxicity was classified and represented across distinct external sources.

### 2.2 Synergy scores and toxicity analysis

**Figure 2.**
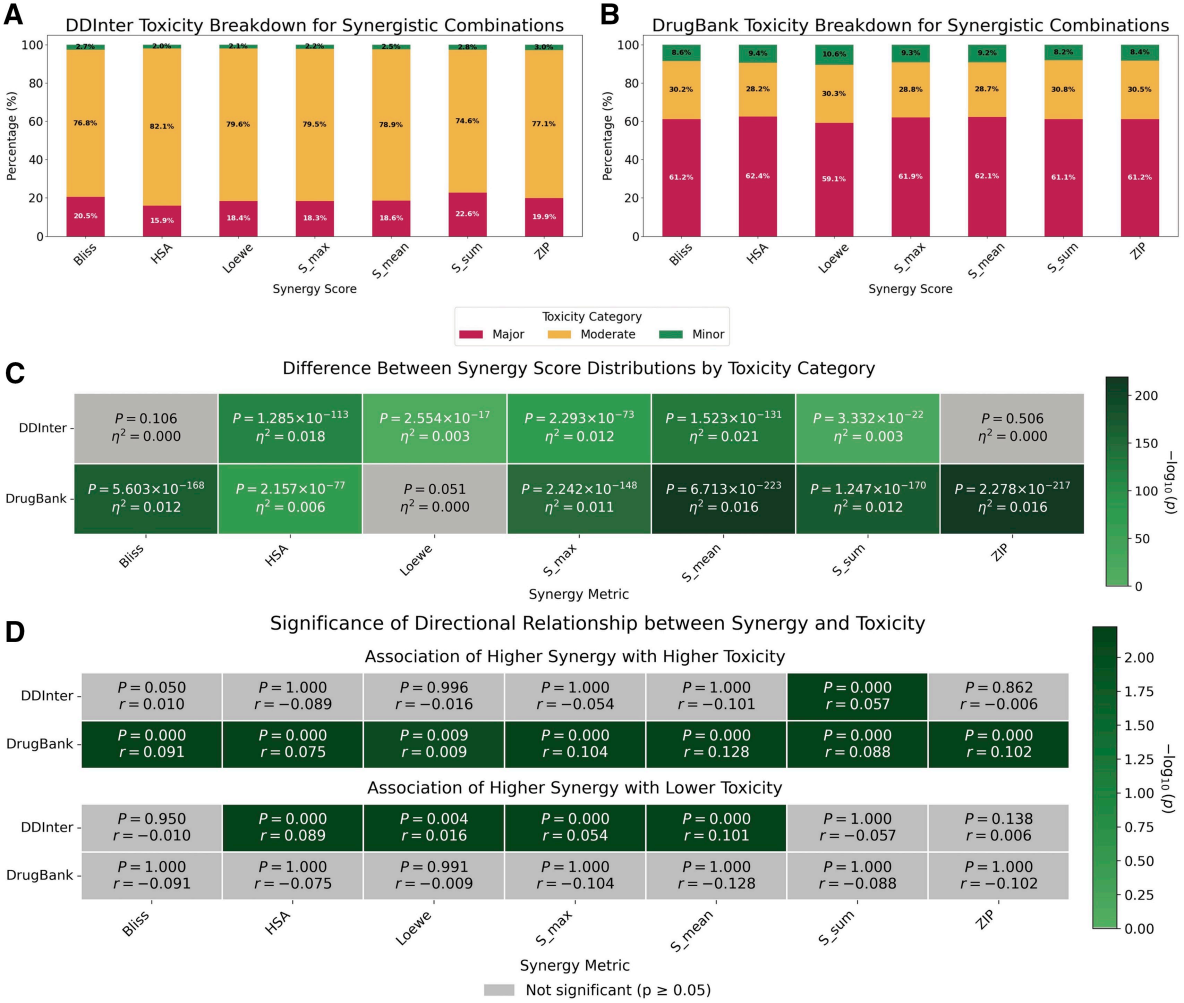
Synergistic drug combinations trend with higher toxicity in DrugBank but not DDInter. Panel (A) and Panel (B) display the proportional distribution of toxicity categories (Minor, Moderate, and Major) for synergistic combinations within the DDInter and DrugBank databases, respectively. Each bar represents a distinct synergy scoring metric. Panel (C) summarizes the results of the Kruskal-Wallis test, determining if there was a statistically significant difference in the distribution of synergy scores among the three toxicity categories (Minor, Moderate, Major) for each metric and database. Each cell reports the *P*-value and the $\eta^{2}$ (eta squared) effect size. Panel (D) illustrates the results of the Jonckheere-Terpstra test, a nonparametric test designed to detect a trend across ordered groups. The test assesses whether synergy scores exhibit a significant increasing (or decreasing) trend corresponding to the ordered toxicity levels (Minor → Moderate → Major). Each cell provides the *P*-value and the *r* (Jonckheere-Terpstra statistic) effect size. Green cells indicate a statistically significant effect (p<0.05). Additional DrugBank synergy score distributions are presented in Figure S3, available as supplementary data at *Bioinformatics* online and DDInter synergy score distributions are presented in Figure S4, available as supplementary data at *Bioinformatics* online.

In the first analysis, we examined whether synergy scores have any relationship with DDI severity. We focused our analysis on five different synergy scores: Bliss, Highest Single Agent (HSA), Loewe, S, and Zero Interaction Potency (ZIP) (Supplementary Methods, available as supplementary data at *Bioinformatics* online). For the S synergy score model, we obtained the three calculated variations: S_max, S_mean, or S_sum. We first characterized the percentages of toxicity categories among synergistic combinations (Fig. 2A and B). We then tested whether drug combination synergy scores were normally distributed using scipy’s normaltest function (Virtanen *et al.* 2020), based on the D’Agostino and Pearson’s test (D’Agostino, 1971, D’Agostino and Pearson, 1973). Each of the seven synergy scores did not appear to follow Gaussian distributions (p≤0.05 where the null hypothesis assumes a sample was drawn from a normal), leading us to use nonparametric tests.

Both DrugBank (Knox *et al.* 2024) and DDInter (Xiong *et al.* 2022) store the DDI information in three categorical measures describing potential interactions between drugs. DrugBank reported the toxicity levels in a numerical format (0,1,2) which was converted to Minor, Moderate, and Major labels (Knox *et al.* 2024). DDInter reported the toxicity using categorical values Minor, Moderate, and Major (Xiong *et al.* 2022). In both cases, Minor describes an interaction that was unlikely to have a significant effect on the patient; Moderate describes an interaction that may cause a negative effect or an exacerbation of a symptom; and Major describes a possible significant or life-threatening interaction. As a result, we split the synergy scores across these three categorical levels to test if the toxicity categories had significant differences in the medians of their synergy score distributions using the Kruskal-Wallis test (via the function scipy.stats.kruskal) (Kruskal and Wallis 1952, Virtanen *et al.* 2020). Due to the varying sample sizes *N* between DrugBank and DDInter, we calculated the effect size $\eta^{2}=H/(N-1)$ based on the Kruskal-Wallis *H*-statistic to determine the direction and magnitude of the difference between toxicity categories. Here, $\eta^{2}$ measures the proportion of variance in the ranks explained by the group membership.

For post-hoc analysis, we applied Dunn’s test (Dunn 1964, Glantz 2012) with Bonferroni correction using scikit_posthocs.posthoc_dunn (Terpilowski 2019) to assess whether there were significant differences in the medians between pairwise categories (i.e. between Major and Moderate, Moderate and Minor, or Major and Minor). We also calculated the Cliff’s Delta δ, a nonparametric effect size for two groups that quantifies the probability that a randomly selected value from one group is larger than a randomly selected value from the other group.

To interrogate whether optimizing for synergy had any bearing on lowering toxicity, we used the Jonckheere-Terpstra test (Terpstra 1952, Jonckheere 1954) to assess the directionality of trends between the toxicity levels and synergy scores. In other words, we tested if optimizing for higher synergy scores was correlated with lower toxicity. Here, we calculated the nonparametric effect size $r=Z/\sqrt{N}$ from the Jonckheere-Terpstra *Z*-statistic, which measures the strength of the monotonic trend between toxicity level and synergy score (scaled by the square root of total sample size *N*). The magnitude of the effect size *r* can be interpreted similarly to a Pearson’s correlation coefficient. First, we tested if the medians of the synergy score distributions increase as toxicity levels decrease. We then computed whether the medians of synergy scores increase as toxicity levels increase. All statistical tests, effect sizes, and *P*-values are present in Fig. 2C and D, Figures S1 and S2, and Table S2, available as supplementary data at *Bioinformatics* online. Box plots for the synergy scores broken down by toxicity level are available in Figures S3–S5, available as supplementary data at *Bioinformatics* online.

### 2.3 Drug target and pathway analysis

In the second component of our analysis, we investigated the mechanisms that underpin toxicity and synergy. We used drug targets and their UniProtIDs from DrugBank (Knox *et al.* 2024). We then mapped the targets to the pathways they belong to in the Reactome database (Milacic *et al.* 2024). We limited our search of Reactome pathways to those in *Homo sapiens*. Reactome stores a hierarchical structure of pathways, so we included two categories of pathway levels when computing the overlap of pathways between two drug targets. The “lowest pathway level” was the set of pathways in Reactome that belongs to the lowest level of the hierarchy (i.e. the bottom child nodes in the Reactome pathway tree). The “all pathway levels” category includes all pathways in Reactome, regardless of where in the hierarchy it belongs. Drug targets, their pathways at the lowest level, and their pathways at all levels were used in the drug target and pathway analysis section.

We first examined whether drug target and pathway overlap was correlated with DDI severity levels. For each dataset (DrugBank and DDInter), we computed the Jaccard Similarity, a metric of overlap (Jaccard 1901, 1912), for all drug combinations for each of these three categories. For example, for each (drug *A*, drug *B*, cell line *C*) triplicate, we find the set of all targets for drug *A*, $T_{A}$, the set of all targets for drug *B*, $T_{B}$, and compute the Jaccard Similarity J(A,B) between the two sets. The Jaccard Similarity was calculated as


(1) $$J(T_{A},T_{B})=\frac{\left|T_{A}\cap T_{B}\right|}{\left|T_{A}\cup T_{B}\right|}.$$

**Figure 3.**
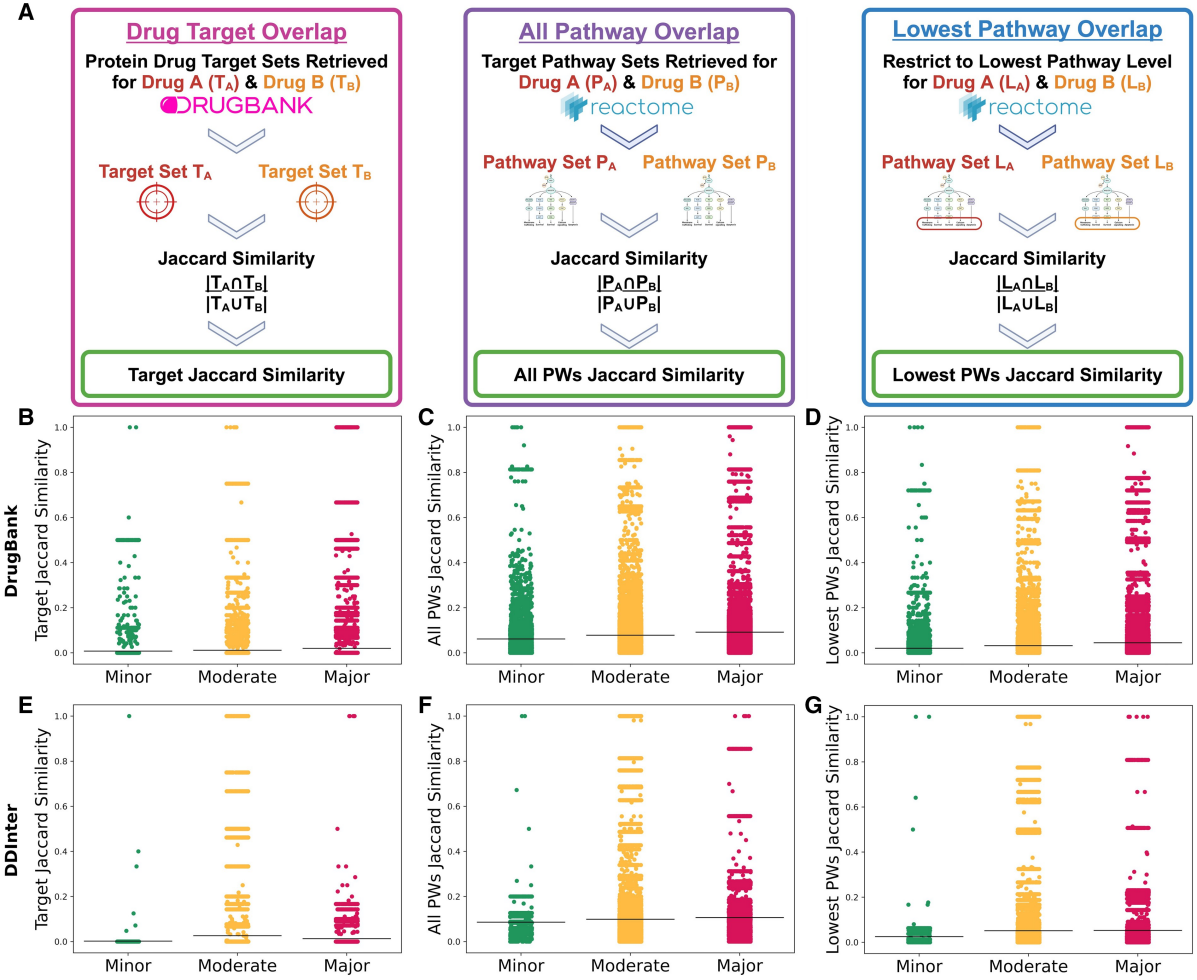
Drug target and pathway overlap metrics do not consistently separate toxicity categories. Panel (A) shows a flow chart illustrating how each of the Target, All Pathways (All PWs), and Lowest Pathways (Lowest PWs) Jaccard Similarities were calculated. The remaining panels are strip plots displaying the distributions of drug combination target and target pathway overlap when split across toxicity categories. The mean of each distribution was denoted by the black horizontal line. All distributions representing the Minor toxicity are in green, the Moderate in yellow, and the Major in red. The top row including panels (B), (C), and (D) used DrugBank toxicity categories, while the bottom row of panels (E), (F), and (G) correspond to DDInter. Panels (B) and (E) show the Jaccard distributions of toxicity categories based on the set of target proteins for each drug in a drug pair. Panels (C) and (F) display the Jaccard Similarity distributions based on all Reactome pathways, while Panels (D) and (G) restrict Reactome to its lowest level pathways.

This process was also applied to the drug targets’ Reactome pathways, resulting in Jaccard Similarity scores for drug targets, the lowest Reactome pathways, and all Reactome pathways. We then tested each of these similarity scores for normality using the D’Agostino and Pearson’s test. Neither DrugBank nor DDinter were normally distributed (p≤0.05), so we used the Kruskal-Wallis test to assess if toxicity categories had significant differences in the medians of the drug target or pathway Jaccard Similarities. We performed post-hoc analysis via Dunn’s test with Bonferroni correction. To determine whether there was a significant ordering of Jaccard Similarity medians when increasing or decreasing the toxicity level, we used the Jonckheere-Terpstra test. We also calculated the effect size for each statistical test to quantify the strength of the relationship between toxicity levels and the database entries. All statistical test outcomes can be found in Table S3, available as supplementary data at *Bioinformatics* online. A schematic depicting the steps for this analysis was presented in Fig. 3A. Strip plots to show the Jaccard Similarity distributions are shown in Fig. 3B–G.

**Figure 4.**
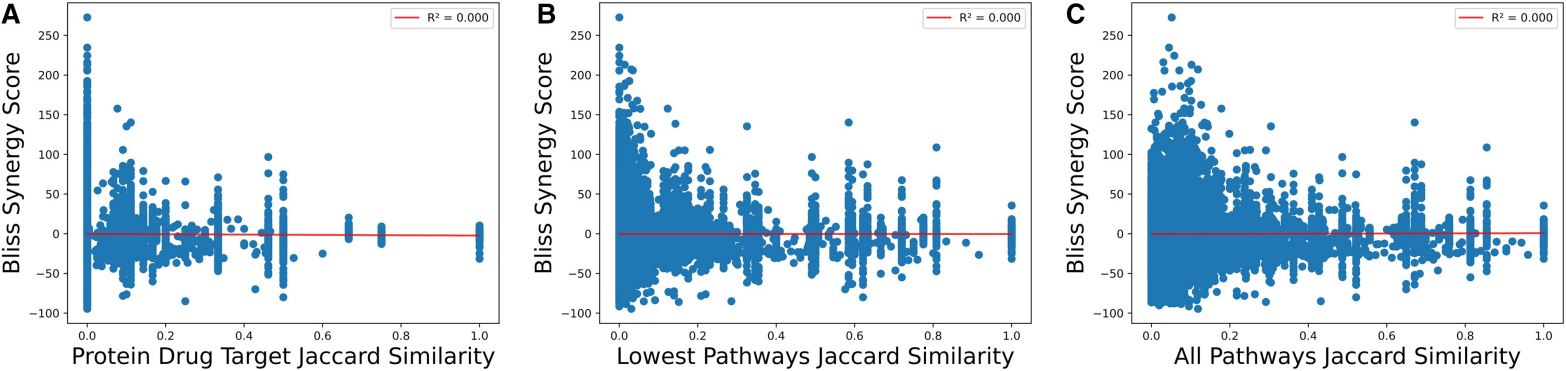
Synergy scores show no correlation with drug target overlap metrics from DrugBank. Scatter plots showing the Bliss synergy score versus three drug target overlap metrics for combinations present in both DrugComb and DrugBank. Panel (A) corresponds to the Jaccard Similarity of drug combinations’ protein targets. Panel (B) shows the Jaccard Similarity of drug target pathways when restricted to the lowest level of Reactome. Panel (C) displays the Jaccard Similarity of drug target pathways at all levels of Reactome. Each plot also contains a red line for the best fit line, with the $R^{2}$ present in the legend, which are all zero. See Figure S6, available as supplementary data at *Bioinformatics* online for all other synergy scores.

We also examined whether there was a correlation between drug target and pathway overlap with synergy scores. We used the same Jaccard Similarity metrics for drug targets, the lowest Reactome pathways, and all pathways to compute a Pearson (Pearson 1895) and Spearman (Spearman 2010) correlation with each of the synergy scoring methods. We then created scatter plots with best fit lines and their R-squared ($R^{2}$) (Wright 1921) value. We completed these steps both for the preprocessed DrugBank and DDInter datasets, which can be found in Fig. 4, Figures 6 and 7, and Table S4, available as supplementary data at *Bioinformatics* online.

### 2.4 Toxicity score analysis

**Figure 5.**
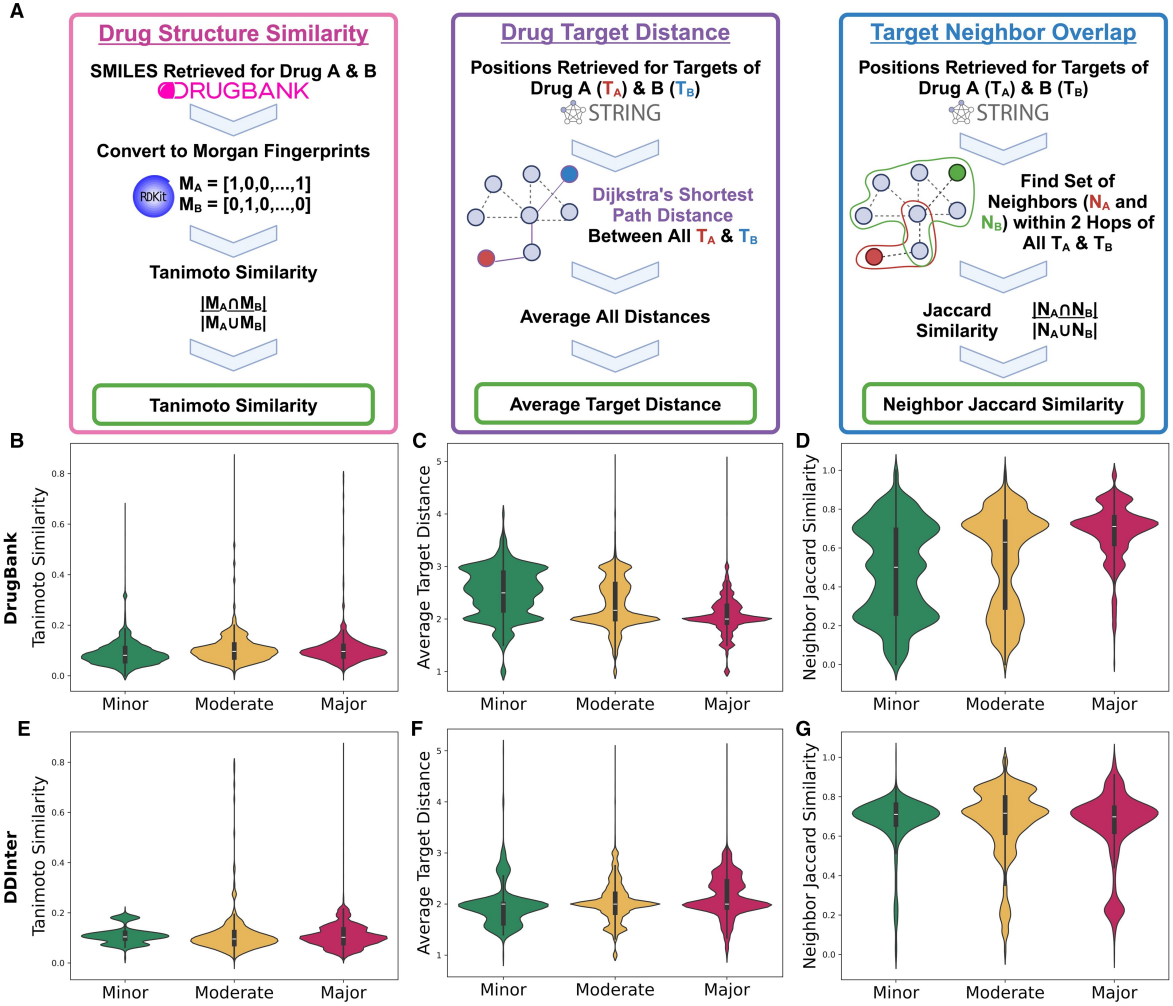
Toxicity metric distributions differ between toxicity categories, but still show large overlaps. Panel (A) contains a schematic illustrating how each toxicity metric was calculated. Panels (B–G) include violin plots displaying the distributions of three toxicity scoring metrics, split by toxicity categories determined by DrugBank or DDInter, all of which have considerable overlap. The mean of each distribution was denoted by the black horizontal line. All distributions representing the Minor toxicity are in green, the Moderate in yellow, and the Major in red. The first row (B–D) corresponds to toxicity categories determined via the DrugBank database. The second row (E–G) corresponds to the toxicity categories determined by the DDInter database. The first column (panels (B) and (E)) shows the Tanimoto Similarity distributions between drug structure Morgan Fingerprint representations in each drug pair. The second column (panels (C) and (F)) uses the average target distance between the sets of drug targets in a drug combination when Dijkstra’s shortest path was applied to the target locations on the STRING PPIN network. Panels (D) and (G), in the last column, displays the distribution of the Jaccard Similarity of the sets of drug targets’ neighbors, within two-hops on the STRING network.

Some cancer drug combination prediction models integrate toxicity scores into their loss functions to balance optimizing for high synergy while minimizing toxicity. We assessed three metrics to test key components of toxicity scores (Fig. 5A). First, some methods use drug structure similarity as a toxicity penalty term (Rafiei *et al.* 2023). To assess this, we retrieved the SMILES (Weininger 1988) string representations for all drug structures from DrugBank. We then used RDKit (Landrum *et al.* 2023) to map the SMILES representations to Morgan Fingerprint 2048-bit vectors (Morgan 1965). For each drug combination (say drug *A* and drug *B*), we used the corresponding Morgan Fingerprint vectors $\left(M_{A},M_{B}\right)$, and calculated the Tanimoto Similarity $T(M_{A},M_{B})$ (Tanimoto 1957). The Tanimoto Similarity is equivalent to the Jaccard Similarity. For bit vectors like Morgan Fingerprint representations, $T(M_{A},M_{B})$ can be expressed as


(2) $$T(M_{A},M_{B})=\frac{M_{A}M_{B}}{|M_{A}{|}^{2}+|M_{B}{|}^{2}-M_{A}M_{B}}$$

Once all Tanimoto Similarities had been calculated, we plotted the distributions of Morgan Fingerprint similarities in violin plots by toxicity categories from both DrugBank and DDInter.

The second metric we examined was the average distance between targets of each drug in a combination. Since toxicity is thought to be due to the overlap of drug target pathways, we investigated whether larger overall distances between drug targets meant lower severity of DDIs. To compute this average target distance score, we used the STRING PPIN database (Szklarczyk *et al.* 2023), limited to known interactions (rather than predicted) in *Homo sapiens*. We used the target UniProt IDs retrieved from DrugBank and converted these to STRING IDs using the UniProt database (Consortium 2024). To find the average target distance for a given drug combination (drug *A* and drug *B*), let $T_{A}=\{t_{A,1},t_{A,2},\ldots ,t_{A,n}\}$ as the set of targets for drug *A* and $T_{B}=\{t_{B,1},t_{B,2},\ldots ,t_{B,m}\}$ as the set of targets for drug *B*. Next, let G=(V,E) represent the STRING PPIN where *V* is the set of proteins and *E* is the set of interactions. Let $d_{G}(u,v)$ represent Dijkstra’s (Dijkstra 1959) shortest path distance between the *u*-th and *v*-th proteins. The average target distance between all respective targets of drugs *A* and *B* can be formulated as:


(3) $$D_{\mathrm{avg}}(T_{A},T_{B})=\frac{1}{\left|T_{A}||T_{B}\right|}\sum_{t_{A}\in T_{A}}\sum_{t_{B}\in T_{B}}d_{G}(t_{A},t_{B}).$$

After computing the average target distance for all drug combinations, we then separated the distance values by either DrugBank or DDInter toxicity categories. We again use violin plots to show the distributions of average target distance between Minor, Moderate, and Major toxicity.

The last toxicity score aggregated drug target neighborhood information via the STRING PPIN (Yang *et al.* 2021, Rafiei *et al.* 2023). Yang *et al.* (2021) used neighborhoods that extended two hops from the original drug target. We evaluated whether the overlap of two hop neighborhoods was correlated with known toxicity. For a drug combination (drug *A* and drug *B*) and their set of drug targets ($T_{A}$ and $T_{B}$), we found the positions of all targets on the STRING graph G=(V,E). For each target $t_{A,i}\in T_{A}$, we found all v∈V, where *v* is within two degrees of $t_{A,i}$, creating a neighborhood set of proteins $N_{A}$. We also found all v∈V that are neighbors for the targets of drug *B*, creating $N_{B}$. To calculate the neighborhood overlap, we then used the Jaccard Similarity of $N_{A}$ and $N_{B}$. As with the other metrics, we separated neighbor Jaccard Similarity by toxicity categories via DrugBank or DDInter and created violin plots of the distributions.

For each of these metrics, we tested for normality via scipy’s normaltest function and determined the distributions were not normal (p≤0.05). Thus, we used the nonparametric tests of the Kruskal-Wallis and Dunn’s with Bonferroni correction to assess whether different toxicity categories had different toxicity scores medians. We then used the Jonckheere-Terpstra test to assess if the toxicity score medians trended with higher or lower toxicity. Corresponding effect size measurements were computed to establish the direction and magnitude of the statistical differences between DrugBank and DDInter.

## 3 Results

### 3.1 Optimizing for synergy scores alone may prioritize toxic combinations

To investigate the relationship between synergy scores and toxicity levels, we integrated synergy scores from the DrugComb dataset (Zagidullin *et al.* 2019) with drug-drug interaction (DDI) severity levels sourced from DrugBank (Knox *et al.* 2024) and DDInter (Xiong *et al.* 2022). This approach allowed us to determine whether current methods optimizing solely for high synergy scores are independent of toxicity or if higher synergy indicates reduced toxicity. First, we explored the percentages of synergistic combinations across each DDI severity level in the post-processed datasets (Fig. 2A and B). It was notable that for all synergy scores, 58.8% of drug combinations are labeled as Major adverse interactions, and 10.8 % of DDIs were classified as Minor toxicity entries by DrugBank. DrugComb is one of the most commonly used cancer drug combination datasets for predicting synergy, and if the dataset is biased too heavily to high toxicity combinations, prediction models may be prioritizing more toxic candidates.

To test if there was a statistically significant difference between the distribution of synergy scores among the three toxicity categories (Minor, Moderate, and Major), we applied the Kruskal-Wallis test. In DrugBank, we found that the majority of synergy metrics show a highly significant overall difference among the toxicity groups (p≪0.05) (Fig. 2C). In DDInter, most synergy scores, HSA ($p=1.285\times 10^{-113}$), S_max ($p=2.293\times 10^{-73}$), S_mean ($p=1.523\times 10^{-131}$), and S_sum ($p=3.332\times 10^{-22}$), also showed significant differences in synergy score distributions across toxicity category, consistent with DrugBank results. In all significant cases, the effect size estimates were small, with $\eta^{2}$ ranging from 0.003 to 0.021. Despite strong statistical evidence of a difference, toxicity grouping accounted for less than 2.1% of the variation in synergy scores, implying that toxicity is a weak determinant and other variables drive synergy outcomes.

To complement this global assessment, we also computed Dunn’s test (with Bonferroni correction) on each of the synergy score distributions to determine if there were significant differences between the medians of each pair of toxicity categories (Figure S1 and Table S2, available as supplementary data at *Bioinformatics* online). In other words, we explored if there were differences in a synergy score’s Major toxicity distribution compared to its Moderate distribution (Major versus Moderate). We also compared all other categories (Moderate versus Minor, Major versus Minor). All synergy scores, except Loewe, demonstrated statistical significance (p≪0.05) in DrugBank, indicating that toxicity and synergy scores are not independent. The effect sizes for the DrugBank dataset were moderate to large in magnitude for the Major versus Minor toxicity comparison, with δ ranging from 0.115 to 0.244, indicating that synergy scores for Major interactions are substantially different from Minor interactions in that particular database. Overall, we observed minimal practical differences between Major and Moderate toxicity synergy score distributions. However, the comparison between Moderate and Minor toxicity was highly dependent on the synergy metric used, showing minimal effects for metrics like HSA and Loewe, but clear, moderate-to-large practical differences for ZIP and S_sum.

Next, we applied the Jonckheere-Terpstra test to assess whether increasing synergy scores trend with lower toxicity Fig. 2D and (Table S2, available as supplementary data at *Bioinformatics* online). In the DrugBank database, we observed the opposite. No synergy scores had a significant association between high synergy and low toxicity (p≥0.05), while all synergy scores were found to have increasing toxicity categories significantly associated with higher synergy scores (p≈0; Table S2, available as supplementary data at *Bioinformatics* online). The nonparametric effect size for the increasing-toxicity trend was consistently small (*r* ranging from 0.009 to 0.128), yet the positive values confirm that synergy scores tend to increase as toxicity increases. This indicates that current methods prioritizing highly synergistic combinations may actually increase the risk of Major DDIs.

### 3.2 Correlation strength between synergy and toxicity is both score and database dependent

Notably, many of our observations are dependent on the specific synergy score or toxicity database used. For example, applying the Kruskal-Wallis test to DDInter toxicity categories revealed three metrics with differing conclusions from DrugBank: Bliss (p=0.106) and ZIP (p=0.506) were not statistically significant in DDInter, while Loewe ($p=2.554\times 10^{-17}$) was significant in DDInter but not in DrugBank (Fig. 2C).

These inconsistencies also appeared within Dunn’s post-hoc tests. DrugBank showed consistently significant differences across all comparisons and metrics, meaning synergy scores strongly differ between toxicity levels (Figure S1 and Table S2, available as supplementary data at *Bioinformatics* online). However, DDInter showed fewer significant differences across all three comparisons, suggesting that synergy scores in DDInter are less systematically associated with toxicity categories. The effect sizes were small-to-moderate (maximum δ≈0.244) and indicated higher synergy in the more toxic groups (Figure S2, available as supplementary data at *Bioinformatics* online).

Additionally, the correlation between higher synergy score and higher toxicity of drug combinations also demonstrated dataset dependence. The S_sum metric, via the Jonckheere-Terpstra tests, was the only synergy score that was significantly associated with the trend of higher synergy correlating with higher toxicity in both databases (Fig. 2D). There was no single synergy score where both datasets agreed that higher synergy scores were significantly associated with lower toxicity.

Across statistical tests, DrugBank showed strong and consistent differences in synergy scores across toxicity categories for nearly all synergy metrics. On the other hand, DDInter displayed weaker and less uniform patterns. This suggested that synergy-toxicity relationships were much clearer in DrugBank than in DDInter. While the relationship between synergy and toxicity appears to be score and dataset dependent, our analyses show that broadly prioritizing synergy alone may not lead to optimally low toxicity combinations.

### 3.3 Comparison between DrugBank and DDInter synergy score distributions

Since the perceived relationship between synergy score and toxicity can be skewed by the database being studied, we investigated the differences between DrugBank and DDInter to understand how this may affect our results. When comparing the distributions of the synergy scores, DrugBank and DDInter have similar means and distributions across both toxicity (Figures S3 and S4, available as supplementary data at *Bioinformatics* online) and synergy scores (Figure S5, available as supplementary data at *Bioinformatics* online). On the other hand, the toxicity categories represent different fractions in the DDInter database, where most synergistic combinations were classified as having Moderate toxicity. In DrugBank, the majority of DDIs were classified as having Major toxicity (Fig. 2A and B). Lastly, the sample sizes varied as well. DrugBank (Knox *et al.* 2024) had about twice as many entries (*N *= 62 728) as DDInter (Xiong *et al.* 2022) (*N *= 29 064), giving DrugBank potentially greater statistical power (see Table S1, available as supplementary data at *Bioinformatics* online). Thus, assessing the magnitude of the effect size across both datasets was important for interpreting the strength of the correlation.

### 3.4 Overlap in drug targets correlates with toxicity but fails to distinguish between toxicity categories

To better understand the biological mechanisms contributing to different DDI severity levels and synergy, we also retrieved drug target and pathway information. Here, we used the Jaccard Similarity to quantify the overlap between a drug combination’s drug targets, pathways, and lowest-level pathways (Fig. 3A). We first used the Kruskal–Wallis test to verify if the medians of the Jaccard Similarity distributions differed when split by toxicity category, which we found to be significant (p<0.05) in all similarity classes and both DrugBank and DDInter (Supplementary Table 3). We then assessed whether there were pairwise differences in similarity distributions using Dunn’s test with Bonferroni correction, and we found all post-hoc analyses to also be significant (p<0.05) (Table S3, available as supplementary data at *Bioinformatics* online). Previous studies have attributed toxicity to high overlap of drug target pathways (Cheng *et al.* 2019, Yang *et al.* 2021, Rafiei *et al.* 2023), so our initial hypothesis was that higher Jaccard Similarity in all classes (drug targets, all Reactome pathways, or Reactome’s lowest pathways) would trend with increasing toxicity levels. To test this, we applied the Jonckheere-Terpstra test and found that the median Jaccard Similarities in all classes were significantly associated with higher toxicity levels (p<0.05) (Table S3, available as supplementary data at *Bioinformatics* online). These findings confirm that overlapping drug targets and their pathways can recapitulate toxicity information in known DDI databases like DrugBank and DDInter. The lowest Reactome pathway similarity (effect size *r* ranging from 0.111 to 0.159) and all Reactome pathways (effect size *r* ranging from 0.122 to 0.129) showed a stronger trend with increasing toxicity compared to drug target similarity (effect size *r* ranging from 0.019 to 0.033). This suggests that pathway level summaries are more indicative of DDI toxicity severity than the individual drug targets.

Because the Jonckheere-Terpstra results indicate that the medians of each overlap distribution increase from Minor toxicity to Major toxicity, one might consider using the Jaccard Similarity metric as a cutoff to distinguish drug combinations’ toxicity. However, our strip plot distributions show considerable overlap for each of the Jaccard Similarity classes (Fig. 3B–G). The distributions do not always show that the Major toxicity category has the most samples with the highest Jaccard Similarity. In fact, there are combinations in all panels that have complete overlap (Jaccard Similarity equal to 1.0) that exist in the Minor category. This indicates that while higher overlap trends with higher toxicity, an overlap metric like Jaccard Similarity does not separate toxicity categories well.

### 3.5 Synergy scores do not correlate with overlap of drug targets and their pathways

In the second aspect of our mechanistic analysis, we also sought to understand whether overlapping drug targets and pathways were correlated with synergy. If certain synergy scores were also associated with drug target overlap, this could account for why some synergy scores trended with higher toxicity. Figure 4 displays scatter plots for DrugBank with the Bliss synergy score on the y-axis and the Jaccard Similarities on the x-axis. The other six synergy scores using the DrugBank dataset were plotted in Figure S6, available as supplementary data at *Bioinformatics* online. Results for DDInter can be found in Figure S7, available as supplementary data at *Bioinformatics* online. Interestingly, none of the results show any discernible correlation, with all $R^{2}$ values near 0. This observation was confirmed when calculating the Pearson and Spearman correlations, which are compiled in Table S4, available as supplementary data at *Bioinformatics* online. All Pearson and Spearman correlation coefficients are near zero, indicating no correlation between synergy scores and drug targets or pathways. This analysis demonstrates that drug target overlap alone was not responsible for the trends we observed among certain synergy scores and toxicity.

### 3.6 Toxicity scoring methods struggle to clearly delineate DDI severity

Finally, we assessed common methods for creating toxicity scores, which have been used to create penalty terms in the loss functions of cancer drug combination prediction models. We computed three metrics: the Tanimoto Similarity of Morgan Fingerprint bit vectors, the average target distance, and the neighbor Jaccard Similarity (Fig. 5). We also performed the Kruskal-Wallis test with Dunn post-hoc analysis (Bonferroni corrected) on each of the toxicity scores. The Jonckheere-Terpstra test was used to evaluate whether an ordered trend exists (i.e. assessing if the score consistently increases or decreases as toxicity becomes more or less severe), with test statistics, *P*-values, and effect sizes being reported in Table S5, available as supplementary data at *Bioinformatics* online.

### 3.7 Morgan fingerprint tanimoto similarity

Morgan Fingerprint Tanimoto Similarity is a chemical similarity measure that calculates the overlap between the Morgan fingerprints of two molecules (i.e., estimates the similarity between the molecular structures of two drugs). We first established through the Kruskal-Wallis test that each score had significantly different medians between toxicity categories, regardless of which database was used. However, the effect size was small in both cases ($\eta^{2}$ = 0.005 and 0.017 in DrugBank and DDInter, respectively). Dunn post-hoc analysis identified significant differences in each of the pairwise DDInter toxicity categories (Dunn’s Test on Major versus Minor DDInter toxicity $p=1.516\times 10^{-3}$, Major to Moderate toxicity $p=1.766\times 10^{-20}$, and Moderate-Minor $p=1.075\times 10^{-13}$). However, not all pairwise categories were significant in the DrugBank toxicity data: the medians of Major and Moderate DrugBank toxicity were not found to be significantly different (p=0.053), although Major to Minor ($p=3.722\times 10^{-216}$) and Moderate to Minor ($p=1.448\times 10^{-208}$) DrugBank toxicity distributions did differ significantly. The effect sizes for all significant pairs were generally small (with the maximum |δ|≤ 0.246). When the Jonckheere-Terpstra test was applied, both DrugBank and DDInter found a significant correlation of increasing Morgan Fingerprint Tanimoto Similarity with increasing toxicity (DrugBank p≈0, DDInter $p=2.622\times 10^{-9}$). These findings agree with the hypothesis that higher drug structure similarity trends with a higher chance of severe DDIs. However, the Tanimoto Similarity distributions have a large amount of overlap between the toxicity categories in both DrugBank and DDInter (Fig. 5B and E). The vast majority of drug combinations also appear to have Tanimoto Similarities between the range of 0 and 0.2.

### 3.8 Average target distance

We next tested whether the closeness of drug targets on a PPIN could correlate well with toxicity levels. We expected that a high average target distance would be correlated with less severe DDIs. We found that, while the DrugBank and DDInter datasets agreed that there were significant differences in the medians of the average target distance distributions between toxicity categories (Kruskal-Wallis and Dunn Test results in Table S5, available as supplementary data at *Bioinformatics* online), there was disagreement when we applied the Jonckheere-Terpstra test. In DrugBank, high target distance significantly trended with lower toxicity (p≈0), but in DDInter, we found the opposite (p≈0). The effect size for the Kruskal-Wallis test was small in DrugBank ($\eta^{2}$ = 0.016) but notably larger in DDInter ($\eta^{2}$ = 0.106). The average target distance as a metric was very dependent on complete knowledge of drug targets and their interactions in a PPIN, so it may be more sensitive to a smaller dataset like DDInter and incomplete target information. Furthermore, our results also show overlapping distributions between toxicity categories (e.g. Fig. 5C and F).

### 3.9 Two-hop neighborhood jaccard similarity

Finally, to assess a drug target-based toxicity score that was less dependent on perfect knowledge of drug targets and their interactions, we considered a two-hop neighborhood Jaccard Similarity analysis. We adopted this measure from the toxicity score used by Yang *et al.* (2021), which aggregated neighbors within two degrees of drug targets. Compared to metrics like the average target distance or drug target overlap, the two-hop neighborhood widened the drug target area from a few specific proteins to a small subnetwork within a PPIN. Assessing the overlap of these subnetworks could protect against the potential pitfalls where incomplete target or interaction information may skew the other metrics. In the neighborhood Jaccard Similarity, we found significant differences in the medians among all toxicity categories (DrugBank p≈0 and DDInter $p=7.618\times 10^{-23}$) for both DDI databases. The effect sizes were relatively small in both DrugBank and DDInter ($\eta^{2}$ = 0.004 and 0.098, respectively). However, there was disagreement (i) in the neighborhood similarity distributions between Major toxicity and Minor toxicity (DrugBank p≈0 versus DDInter p=0.830) and (ii) in whether higher neighborhood similarity was associated with higher toxicity (DrugBank Jonckheere-Terpstra Increasing Toxicity p≈0) or lower toxicity (DDInter Jonckheere-Terpstra Decreasing Toxicity $p=2.220\times 10^{-16}$). Furthermore, our results again show a considerable amount of overlap between distributions (e.g. Fig. 5D and G).

## 4 Discussion

In this study, we explored whether current metrics of synergy and toxicity are well correlated with clinical toxicity in cancer drug combinations, addressing a critical validation need in current computational approaches to combination therapy development. Our findings reveal important limitations in how toxicity is currently incorporated into synergy prediction models. While the field has largely focused on optimizing synergy scores as a primary metric for identifying promising drug combinations, our analysis suggests this may overlook crucial toxicity considerations that impact clinical viability. The complexity of drug-drug interactions (DDIs) extends beyond what current target overlap and structural similarity metrics can capture, highlighting the need for more comprehensive and validated toxicity measurements. These results emphasize the importance of balancing efficacy with safety in a more nuanced and data-driven manner.

First, when examining the DrugComb database, we found a substantial portion of synergistic combinations associated with Major adverse DDIs—even in the most conservative toxicity classification from DDInter, approximately 20% of synergistic combinations showed high toxicity. Our statistical analysis confirmed that synergy scores significantly correlate with increased toxicity levels, showing that synergy and toxicity are not strictly independent. This is especially apparent in the DrugBank data. Still, while the correlation between synergy scores and toxicity was statistically significant, the strength of the relationship is weak. Toxicity was correlated to synergy, but it was far from the only or most dominant factor. These findings suggest an important caution: optimization strategies focused solely on synergy may inadvertently select for combinations with higher likelihood of dangerous drug interactions. Notably, in our study, the relationship between synergy and toxicity varies depending on which synergy scoring method was used, highlighting the critical importance of carefully selecting appropriate synergy metrics when evaluating potential combination therapies.

Our findings also showed to be database dependent: results with DDInter suggest a much weaker and less ordered relationship between synergy and toxicity than DrugBank. This disagreement may be a result of dataset curation. DrugBank’s pipeline prioritizes mechanistic accuracy and consistent clinical validation, producing stable synergy-toxicity relationships across metrics (Knox *et al.* 2024). Here, an automated natural language process (NLP) pipeline combined with a manual expert review is used to extract DDI information from regulatory databases, clinical trial registries, product monographs, and peer-reviewed literature. DrugBank also provides more frequent updates to be a comprehensive resource combining detailed chemical, pharmacological, and target protein data, linking each interaction record to an explicit mechanism and severity. The observed bias towards Major toxicity-related drug combinations in DrugBank may be partially due to the regulatory emphasis, as Major interactions are required by law to be disclosed. Additionally, DrugBank’s curation guidelines are designed to be conservative in their assessments, aiming to be clinically precautionary (Diadkina 2025). While DDInter also uses an NLP pipeline followed by manual pharmacist verification, it is more qualitative and clinically focused than DrugBank, using PubMed and FDA-approved drug labels as its primary source (Xiong *et al.* 2022). DDInter’s broader but less standardized curation potentially introduces heterogeneity that weakens or reverses apparent correlations between synergy and toxicity. DDInter toxicity annotations also rely heavily on (i) the phrasing of the severity definition in the source literature and (ii) the variability in how different databases rate interactions. DDInter excludes “unknown” risks to reduce alert fatigue, which may skew the dataset toward interactions that have clear, moderate-to-severe annotations as shown by the large percentage of Moderate toxicity levels. Due to the difference in size, statistical comparisons across the databases should prioritize effect size over *P*-values, as DrugBank’s larger sample size naturally afford it greater power due to smaller standard errors. This potentially leads DrugBank to have more statistically significant results even for subtle effects and differences between categories. These curation differences may explain why DrugBank consistently showed stronger and more coherent trends linking synergy to toxicity, whereas DDInter produced mixed results.

Our mechanistic investigation into drug-drug interactions revealed nuanced relationships between molecular features, toxicity, and synergy. While we found minimal correlation between synergy scores and either drug target or pathway overlap, we confirmed significant differences in Jaccard Similarities across toxicity categories. The Jonckheere-Terpstra tests verified a clear trend: higher target and pathway overlap consistently correlated with increased toxicity severity across both DrugBank and DDInter databases. This supports the existing understanding that shared targets increase toxic interaction risks. However, our distribution analysis revealed important limitations: some combinations with complete target overlap still exhibited only Minor toxicity, demonstrating that overlap metrics alone cannot definitively predict toxicity outcomes. Taken together, our findings suggest that while target and pathway overlap metrics provide valuable insights for toxicity assessment, they appear to have limited utility as predictors of drug synergy.

We evaluated three approaches to toxicity penalties and identified significant limitations with each method. Morgan Fingerprint Tanimoto Similarity consistently correlated with toxicity across both databases, with higher structural similarity trending toward greater toxicity severity. However, its practical utility as a standalone predictor was limited by substantial overlap in distributions between toxicity categories and the narrow range of observed values (mostly 0–0.2). Our analysis of average target distance revealed concerning inconsistencies between DrugBank and DDInter, highlighting database-dependent variability. Similarly, two-hop neighborhood Jaccard Similarity showed significant distributional differences across toxicity categories but with contradictory directional trends between databases. These inconsistencies highlight a fundamental challenge in developing reliable toxicity penalties for synergy prediction models. While these metrics capture some biologically relevant information, none provides sufficiently robust discrimination between toxicity categories to function effectively as a standalone penalty in computational models. This emphasizes the need for integrative approaches that combine multiple toxicity indicators rather than relying on any single structural or network-based metric.

This study has several limitations, the most striking of which was the dependence on known DDI datasets. Throughout the first and third components of our analyses, we often found conflicting determinations depending on the DDI database used. In the analysis examining synergy scores and toxicity categories, no single synergy metric maintained fully consistent relationships across both databases. Additionally, the drug target-based toxicity scores (average target distance and neighborhood Jaccard Similarity) concluded opposite trends depending on whether DrugBank or DDInter was used. These contradictory findings emphasize that these results are highly dependent on the specific toxicity database employed. Additionally, the nature, screening protocols, and chemical diversity of the drug combinations in DDInter differ substantially from DrugBank, leading to less consistent associations between simple toxicity classifications and synergy output. This also indicates a broader need for agreement between known DDI databases, which is especially important for machine learning methods that make predictions while leveraging prior knowledge. While one source of disparity may be due DrugBank’s more up-to-date curation and large difference in sheer size, future research ought to interrogate the divergence between the two datasets to provide researchers a better understanding of the advantages or biases of using one database over another. Lastly, the availability of high-throughput drug combination toxicity screens on healthy control cells could allow future methods to ground their toxicity penalties in real experimental data.

In conclusion, our study highlights a critical gap in current approaches to cancer drug combination prediction: the inadequate integration of toxicity considerations in synergy-based models. Our findings demonstrate that synergy scores alone may inadvertently bias selection toward more toxic combinations, while common toxicity metrics show inconsistent correlation with clinical adverse effects across different databases. These results underscore the complexity of drug-drug interactions and the limitations of current computational approaches that rely on simplified molecular features or network metrics. Moving forward, we advocate for the development of more comprehensive models that explicitly balance efficacy with safety, incorporating multiple complementary toxicity indicators rather than single metrics. Additionally, we emphasize the urgent need for standardized, high-quality combinatorial toxicity data to support these efforts. Ultimately, we hope this work provides a foundation for more clinically relevant computational methods that can accelerate the discovery of effective cancer combination therapies while prioritizing patient safety.

## Supplementary Material

btag007_Supplementary_Data

## Data Availability

The code written for this project is available at https://github.com/amw14/toxicity-cancer-drug-combination. The RDKit package used to retrieve the Morgan Fingerprint representations of all drug structures was installed using the 2024.09.05 documentation (https://www.rdkit.org/docs/GettingStartedInPython.html). The DrugComb database is publicly available to download at https://drugcomb.fimm.fi/. Version 1.5 was used for this study. For toxicity data, we downloaded the DrugBank (version 5.1.12) including the clinical data under an academic license, which can be found at https://go.drugbank.com/. We also used DDInter, where we downloaded all files in April 2024 from https://ddinter.scbdd.com/download/. Reactome was accessed in December 2024 to map UniProt IDs to human pathways both in the lowest level of pathway as well as all pathways from https://reactome.org/download-data. The STRING PPIN database at https://stringdb-downloads.org/download/protein.physical.links.detailed.v12.0/9606.protein.physical.links.detailed.v12.0.txt.gz was limited to the known interactions in *Homo sapiens*, using version 12.0.
