# Supplementary material for: Characterizing clinical toxicity in cancer combination therapies
Source: Bioinformatics. 2026 Jan 14;42(2):btag007. doi: 10.1093/bioinformatics/btag007 (PMC12865850; doi:10.1093/bioinformatics/btag007)
Supplement: btag007_Supplementary_Data [file btag007_supplementary_data.pdf]

# Supplementary Material to "Characterizing Clinical Toxicity in Cancer Combination Therapies"

This document contains supplementary methods, figures, and tables supporting the analyses presented in the manuscript, "Characterizing Clinical Toxicity in Cancer Combination Therapies". Each figure and table is accompanied by a legend describing the analysis performed and key observations.

## Supplementary Methods

### Clinical drug-drug interaction data

We used two different databases containing information about known DDI data. Information on all database versions and where to access them can be found in the section below entitled "Data and Code Availability". Our primary source of toxicity information was DrugBank, where we retrieved over 1.4 million DDIs under an academic license [1]. These data included the name of each drug in the drug pair, the drug targets for each drug, and the severity level of the DDI (encoded as either 0, 1, or 2 depending on whether the severity was minor, moderate, or major, respectively). We chose DrugBank as our primary dataset due to its more comprehensive size and frequent updates. To assess the reproducibility of our results, we also conducted the same set of analyses using DDInter, which contains 160,235 DDIs [2]. DDInter represented its DDIs by mapping drug pairs to one out of three severity levels (Minor, Moderate, and Major). For consistency and ease of comparison between databases, we converted DrugBank's 0, 1, and 2 severity values to Minor, Moderate, and Major.

### Cancer drug combination synergy data

To evaluate the relationship between synergy scores and the severity of toxicity, we used the cancer drug combination synergy scores in DrugComb [3]. We chose DrugComb for its unification of several pairwise drug combination datasets [4–7] and synergy scoring methodologies. All synergy scores in DrugComb were calculated by assessing the degree of deviation of an observed response from the expected effect of the "additive" interaction. This meant that a synergy score of less than zero is antagonistic, equal to zero is additive, and greater than zero is synergistic. DrugComb reported five distinct models of synergy scores that formulate additivity differently: Bliss, Highest Single Agent (HSA), Loewe, S, and Zero Interaction Potency (ZIP). The S synergy score model has three variations in how it was calculated: S_max, S_mean, or S_sum. Our study included all seven synergy scores. We provide the formulas for each synergy score below, which can also be found on the DrugComb documentation [3, 8, 9].

Let $S_{model}$ represent the synergy score as calculated by a model (i.e., one of Bliss, HSA, Loewe, ZIP, max, mean, or sum). Let $\mathcal{D}$ be the set of drugs and $R_A$ be the measured response of individual drug $A \in \mathcal{D}$. We defined $E_{AB}$ as the measured combination effect between drugs $\{A, B\} \in \mathcal{D}$.

**Bliss.** The Bliss Independence Model assumes that drugs act independently through different mechanisms. Namely,

$$S_{\text{Bliss}} = E_{AB} - (R_A + R_B - R_A R_B)\,. \tag{1}$$

Although the Bliss synergy score is summed over dose-response relationships, it offers an intuitive interpretation of what is considered ‘greater than additive’ activity.

**HSA.** The Highest Single Agent (HSA) model is the most simplistic synergy score. HSA calculates the difference between combination effects and the most effective single drug

$$S_{\text{HSA}} = E_{AB} - \max(R_A, R_B). \tag{2}$$

While straightforward to implement and interpret, HSA is more permissive in identifying synergy and also does not consider dose-response curves, potentially overestimating synergistic effects in many scenarios.

**Loewe.** The Loewe Additivity Model assumes that the combined effect of two drugs can be predicted if the doses of each drug are adjusted to account for their relative potencies

$$S_{\text{Loewe}} = E_{AB} - \mu_{AB}. \tag{3}$$

Let concentrations of drugs $A$ and drug $B$ be denoted by $C_A$ and $C_B$ , respectively. The expected effect $\mu_{AB}$ for a given drug combination $AB$ must satisfy the following

$$\frac{C_A}{C_A^*} + \frac{C_B}{C_B^*} = 1 \tag{4}$$

where $C_A^*$ and $C_B^*$ are the doses of drug $A$ and $B$ that produce the same effect individually as they do in combination. To better understand $C_A^*$ and $C_B^*$, we can substitute them by using dose-response curves described by four parameter log-logistic curves. The condition that the drug combination must satisfy then becomes the following

$$C_A \left[ m_A \left( \frac{\mu_{AB} - E_{A,\min}}{E_{A,\max} - \mu_{AB}} \right)^{\frac{1}{\lambda_A}} \right]^{-1} + C_B \left[ m_B (\frac{\mu_{AB} - E_{B,\min}}{E_{B,\max} - \mu_{AB}})^{\frac{1}{\lambda_B}} \right]^{-1} = 1 \tag{5}$$

where $m_A$ and $m_B$ are the half-maximal inhibitory concentrations for drugs $A$ and $B$ respectively, the values $\{E_{A,\min}, E_{A,\max}\} \in [0, 1]$ are the minimal and maximal effects of the drug, and $\lambda_A$ and $\lambda_B$ are the sigmoidicity shape parameters of the dose response curves. Unlike the previous scores, the Loewe synergy score incorporates dose-response relationships of individual drugs, but is more computationally intensive.

**ZIP.** The Zero Interaction Potency (ZIP) score combines the Bliss and Loewe models by calculating the expected combination effect of two drugs under the assumption that they do not potentiate each other. Namely,

$$S_{\text{ZIP}} = E_{AB} - \beta_{AB} \tag{6}$$

where the expected effect $\beta_{AB}$ for a drug combination $AB$ is given by the following

$$\beta_{AB} = \left( \frac{C_A/m_A}{1 + C_A/m_A} \right)^{\lambda_A} + \left( \frac{C_B/m_B}{1 + C_B/m_B} \right)^{\lambda_B} - \left( \frac{C_A/m_A}{1 + C_A/m_A} \right)^{\lambda_A} \left( \frac{C_B/m_B}{1 + C_B/m_B} \right)^{\lambda_B}. \tag{7}$$

This unified approach addresses the limitations of both parent models but is more complicated to interpret. The ZIP score also requires comprehensive dose-response matrices.

**S Synergy Scores** The S scores are based off of a Combination Sensitivity Score (CSS) that integrates dose response curves to determine the sensitivity of a drug combination. The S scores are calculated as deviations of the observed drug combination effect from the expected effect if the drugs do not interact. Namely,

$$\text{S\_max} = \text{CSS} - \max(\text{AUC}_A, \text{AUC}_B) \tag{8}$$

$$\text{S\_mean} = \text{CSS} - \text{mean}(\text{AUC}_A, \text{AUC}_B) \tag{9}$$

$$\text{S\_sum} = \text{CSS} - \text{sum}(\text{AUC}_A, \text{AUC}_B) \tag{10}$$

where $\text{AUC}_A$ and $\text{AUC}_B$ are areas under the monotherapy dose-response curves. Each S score uses a different reference model: S_max uses the effect of the most potent single drug (similar to HSA), S_mean uses the average effect of individual drugs, and S_sum uses the sum of individual effects.

## Data preprocessing

We used the following procedures to preprocess the data used in this study. First, we focused our study on cancer drug combinations from DrugComb where all seven synergy scores were available. For reproducibility, we then filtered the DrugComb combinations to create two separate datasets: one for the combinations with known toxicity levels in DrugBank, another for DDInter. For both datasets, we then also limited the combinations to drugs that had known drug target information (via DrugBank) and known UniProt IDs for mapping to the Reactome pathways and STRING protein-protein interaction network (PPIN). When all filtering was finished using the DrugBank toxicity levels, there were 526 unique drugs, 53,516 unique (drug $A$, drug $B$, cell line $C$) triplicates invariant to order of (drug $A$, drug $B$) versus (drug $B$, drug $A$), and 186 unique cell lines. Using the DDInter toxicity data, the end result of preprocessing left 331 drugs, 23,415 unique drug-drug-cell line triplicates, and 149 cell lines.

# Supplementary Figures

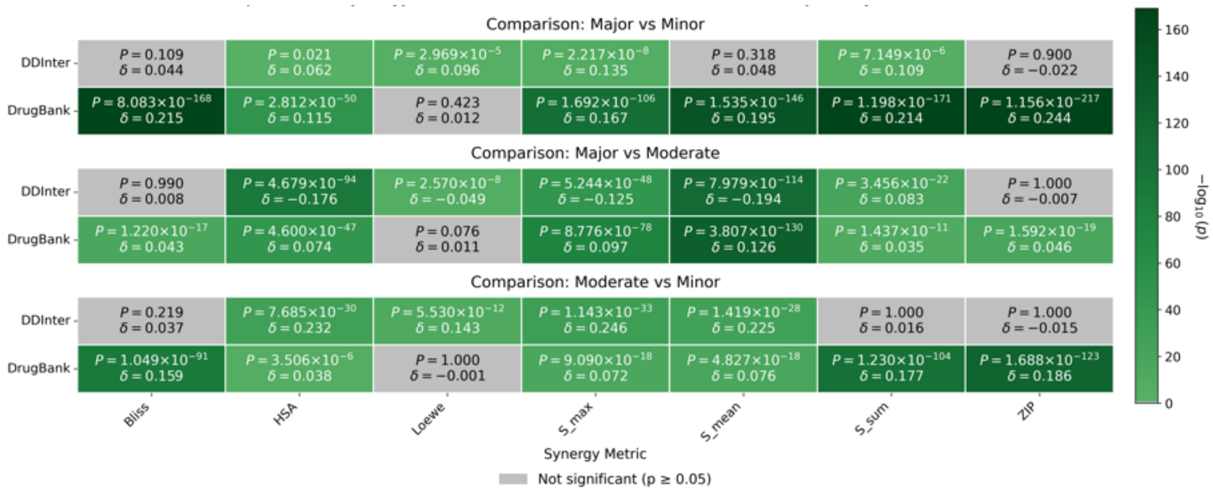


**Supplementary Figure 1. Comparison of synergy score distributions between pairwise toxicity categories.** The figure presents the results of Dunn's post-hoc test, which compares the synergy score distributions between all pairwise toxicity groups (Minor versus Moderate, Minor versus Major, and Moderate versus Major). Each cell reports the adjusted $p$-value (corrected for multiple comparisons) and Cohen's $d$ effect size.

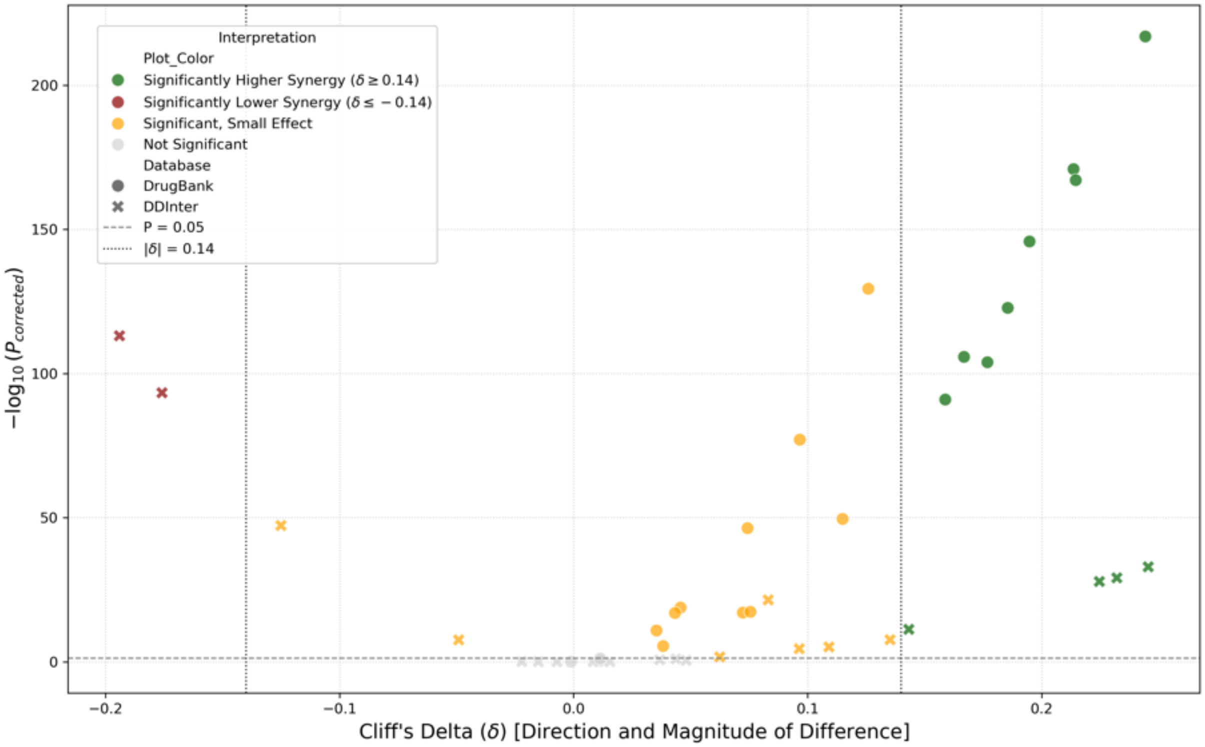


**Supplementary Figure 2. Volcano plot of Dunn's post-hoc test results for pairwise toxicity comparisons.** Volcano plot showing the statistical significance with -$\log_{10}(P_{\text{corrected}})$ on the y-axis versus the magnitude and direction of difference via Cliff's Delta ($\delta$) for all pairwise toxicity comparisons on the x-axis. The plot demonstrates that most significant effects (above the corrected $P < 0.05$ threshold) show higher synergy in the more toxic group ($\delta \geq 0.14$; top right). However, the points in the top-left quadrant (dark red) confirm isolated cases where significantly lower synergy corresponds to increased toxicity ($\delta \leq$ -0.14).

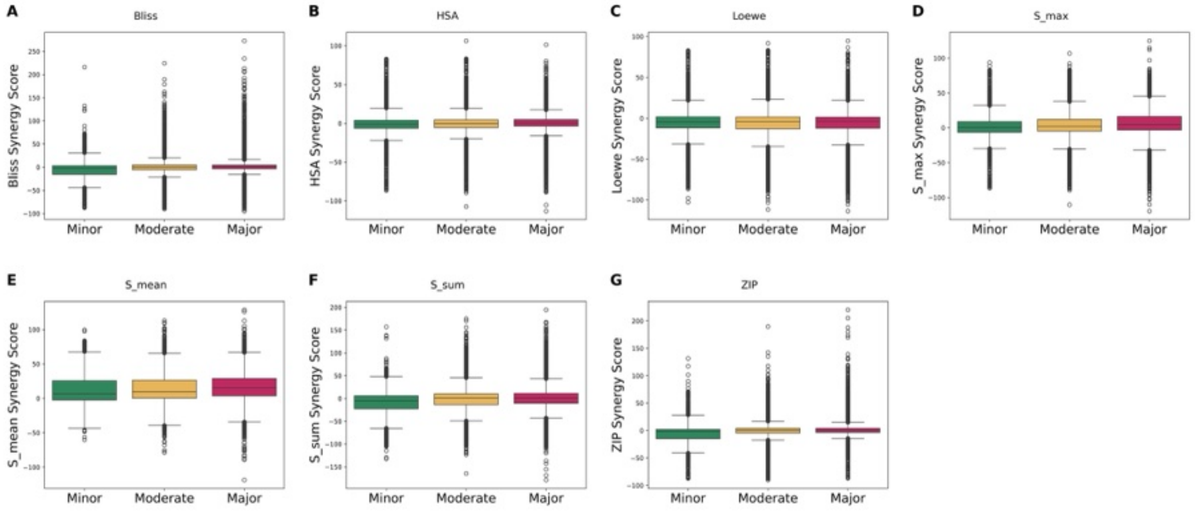


**Supplementary Figure 3. Synergy score distributions show overlap between DrugBank toxicity categories.** Strip plots showing the distributions of drug combination synergy scores when split across toxicity categories, the mean of each distribution is denoted by the black horizontal line. All distributions representing the Minor toxicity are in green, the Moderate in yellow, and the Major in red. Each panel represents a different synergy score: the **(A)** Bliss **(B)** HSA, **(C)** Loewe, **(D)** S_max, **(E)** S_mean, **(F)** S_sum, and **(G)** ZIP Synergy Scores.

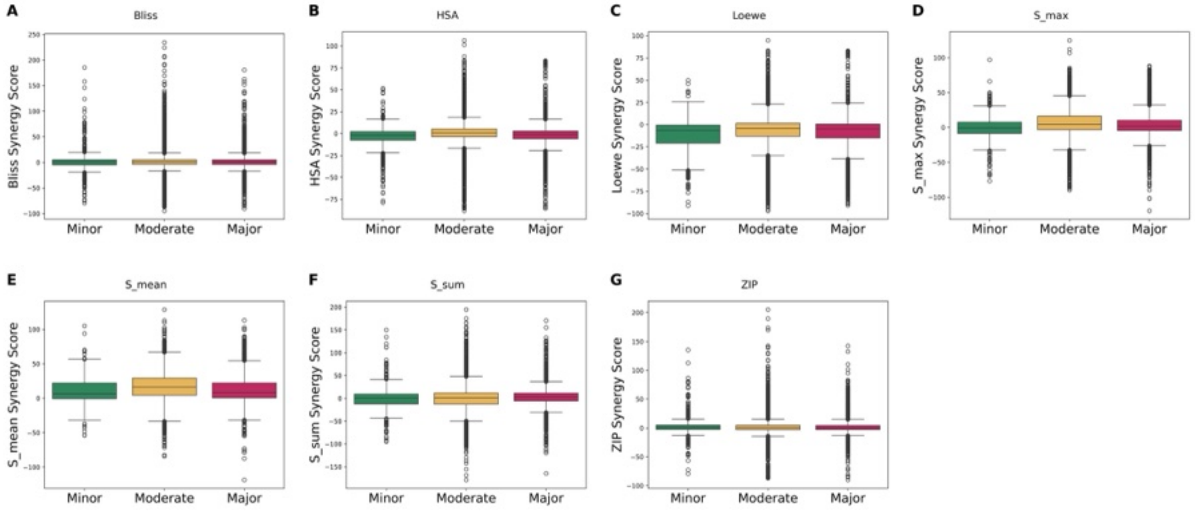


**Supplementary Figure 4. Synergy score distributions by DDInter toxicity category.** Strip plots showing the distributions of drug combination synergy scores when split across toxicity categories, the mean of each distribution is denoted by the black horizontal line. All distributions representing the Minor toxicity are in green, the Moderate in yellow, and the Major in red. Each panel represents a different synergy score: **(A)** the Bliss synergy score, **(B)** HSA, **(C)** Loewe, **(D)** S_max, **(E)** S_mean, **(F)** S_sum, and **(G)** ZIP Synergy Scores.

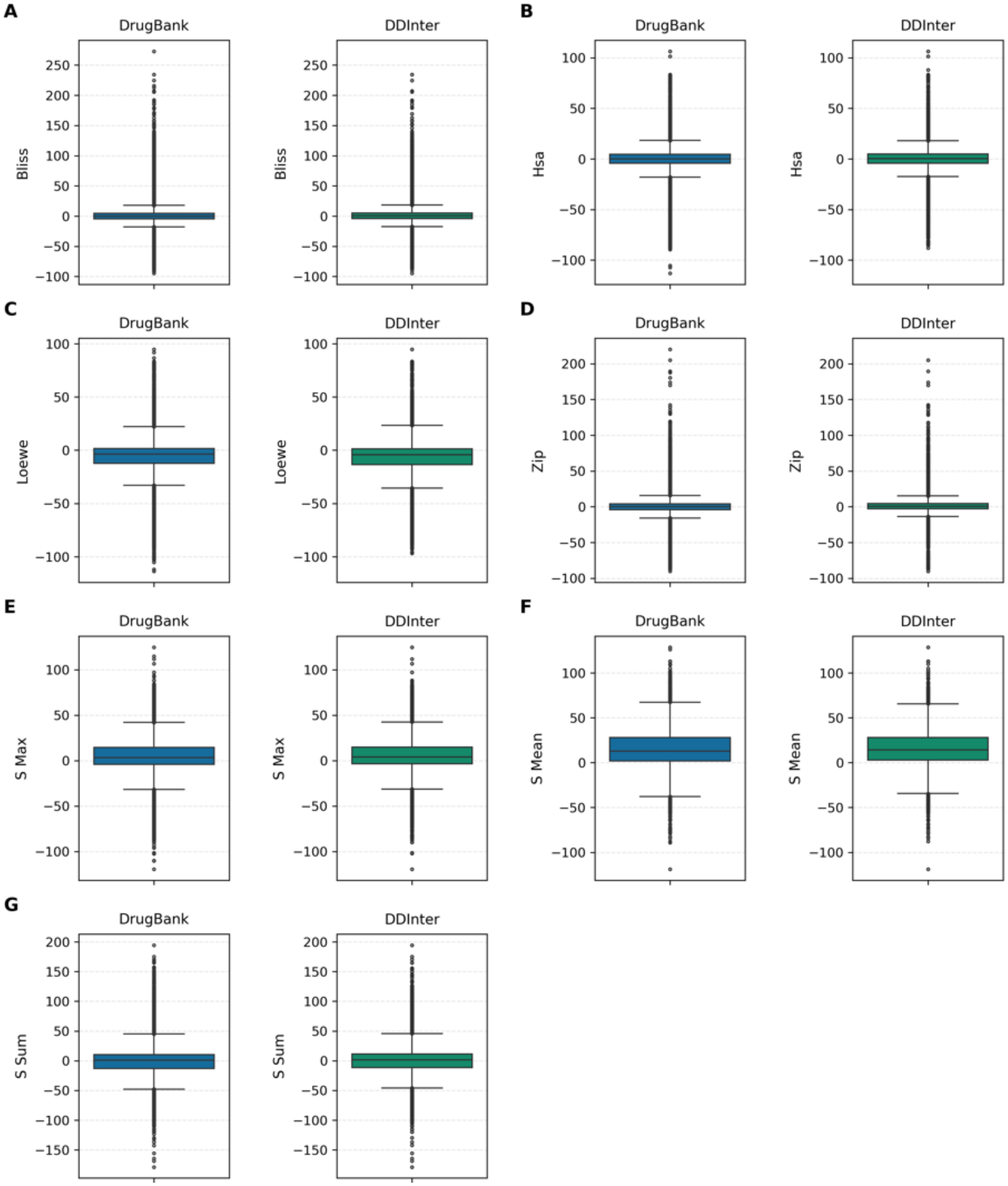


**Supplementary Figure 5. Distributions of synergy scores across DDInter and DrugBank.** Box plots showing the distributions of drug combination synergy scores, where the mean of each distribution is denoted by the black horizontal line. All distributions representing DDInter toxicity are in green and DrugBank is in red. Each panel represents a different synergy score: **(A)** the Bliss synergy score, **(B)** HSA, **(C)** Loewe, **(D)** ZIP, **(E)** S_max, **(F)** S_mean, and **(G)** S_sum.

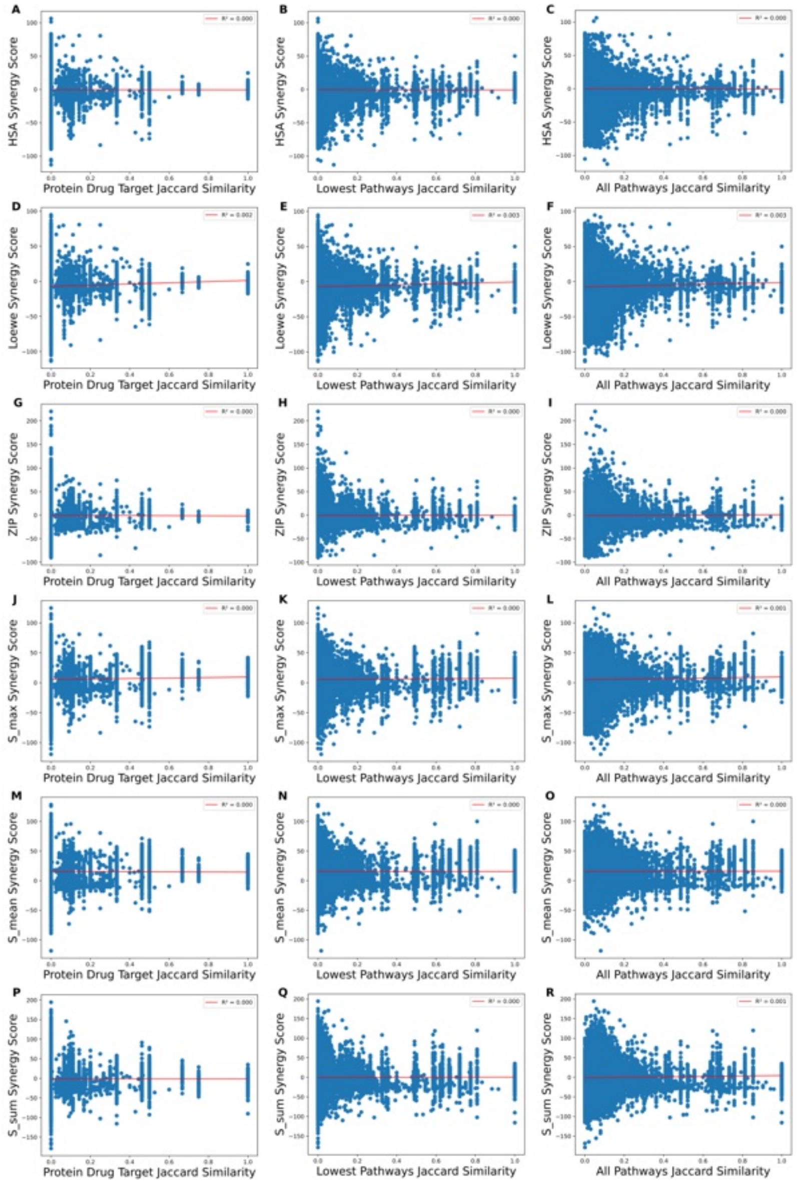


**Supplementary Figure 6. Synergy scores show no correlation with drug target overlap metrics from DrugBank.** Scatter plots showing the relationships between remaining synergy scoring metrics and drug combination target overlaps for drug combinations present in both DrugComb and DrugBank. Each plot also contains a red line for the best fit line, with the $R^2$ present in the legend. Each row corresponds to a different synergy scoring method, and each columns corresponds to a different drug combination target metric. Rows starting with **(A)**, **(D)**, **(G)**, **(J)**, **(M)**, and **(P)** correspond to the HSA, Loewe, ZIP, S_max, S_mean, and S_sum synergy scores. Columns starting with **(A)**, **(B)**, and **(C)** correspond to the Jaccard Similarity of drug combinations' protein targets, pathways when restricted to the lowest level of Reactome, and all pathways of Reactome.

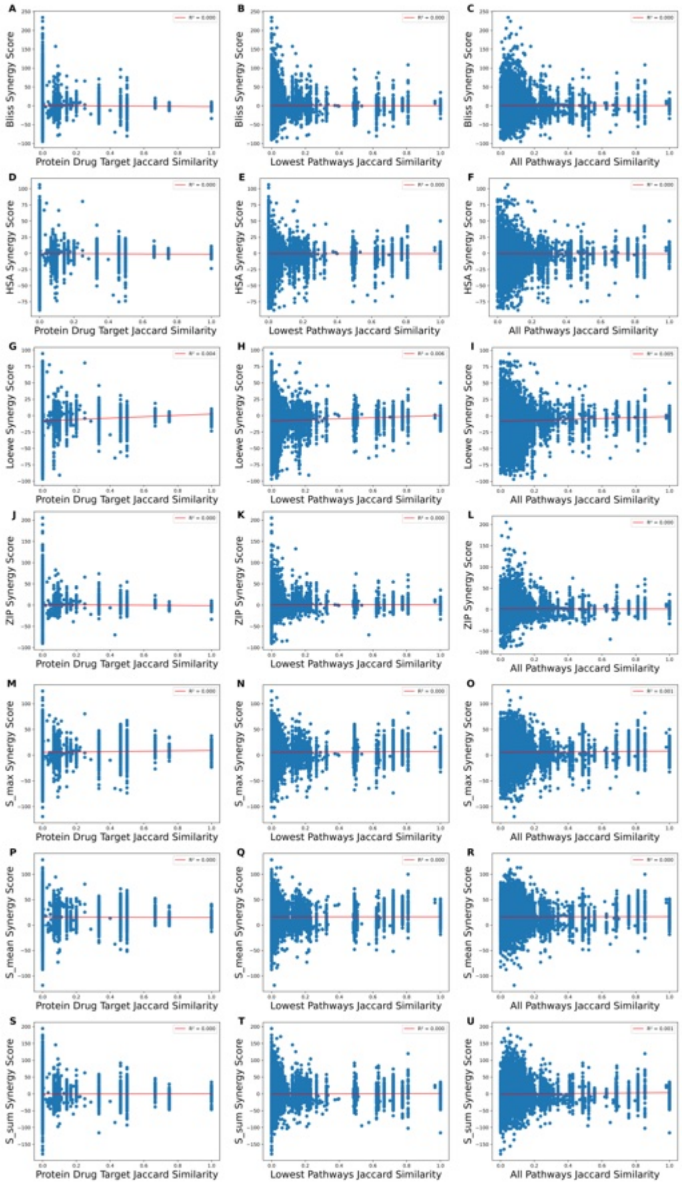


**Supplementary Figure 7. Synergy scores show no correlation with drug target overlap metrics from DDInter.** Scatter plots showing the relationships between synergy scoring metrics and drug combination target overlaps for drug combinations present in both DrugComb and DDInter. Each plot also contains a red line for the best fit line, with the $R^2$ present in the legend. Each row corresponds to a different synergy scoring method, and each columns corresponds to a different drug combination target metric. Rows starting with **(A)**, **(D)**, **(G)**, **(J)**, **(M)**, **(P)**, and **(S)** correspond to the Bliss, HSA, Loewe, ZIP, S_max, S_mean, and S_sum synergy scores. Columns starting with **(A)**, **(B)**, and **(C)** correspond to the Jaccard Similarity of drug combinations' protein targets, pathways when restricted to the lowest level of Reactome, and all pathways of Reactome.

# Supplementary Tables

**Supplementary Table 1. Comparison of summary statistics between the post-processed DrugBank and DDInter databases.**

| Metric | DrugBank | DDInter |
| --- | --- | --- |
| Total Entries | 62728 | 29064 |
| Major Toxicity Entries | 36864 (58.8%) | 5776 (19.9%) |
| Moderate Toxicity Entries | 19074 (30.4%) | 22457 (77.3%) |
| Minor Toxicity Entries | 6790 (10.8%) | 831 (2.9%) |
| Unique Drugs | 526 | 331 |
| Unique Major Toxicity Pairs | 36864 | 5776 |
| Unique Moderate Toxicity Pairs | 19074 | 22457 |
| Unique Minor Toxicity Pairs | 6790 | 831 |
| Number of Cell Lines | 186 | 149 |

**Supplementary Table 2. Statistical analysis of synergy scores by toxicity category.** Results of statistical analysis assessing the relationship between synergy score distributions and known DDI severity categories. Each section corresponds to a different synergy scoring method: Bliss, HSA, Loewe, ZIP, S_max, S_mean, or S_sum. The results for the Kruskal-Wallis, Dunn with Bonferroni correction, and Jonckheere-Terpstra tests are included for both the DrugBank and DDInter datasets. The "Jonckheere-Terpestra: Increasing Toxicity" test assesses whether the distribution of the Jaccard Similarity increases when toxicity categories become more severe, while the "Jonckheere-Terpstra: Decreasing Toxicity" test evaluates if the Jaccard Similarity distributions increase when the toxicity categories decrease in severity.

| **Synergy Score** | **Test** | **DrugBank** | | **DDInter** | |
|---|---|---|---|---|---|
| | | **Test Statistic** | ***P*-value (Effect Size)** | **Test Statistic** | ***P*-value (Effect Size)** |
| **Bliss** | Kruskal-Wallis | $7.702 \times 10^{2}$ | $5.603 \times 10^{-168}$ ($\eta^2 = +0.012$) | 4.491 | 0.106 ($\eta^2 = +0.000$) |
| | Dunn: Major/Minor | - | $8.083 \times 10^{-168}$ ($\delta = +0.215$) | - | 0.109 ($\delta = +0.044$) |
| | Dunn: Major/Moderate | - | $1.220 \times 10^{-17}$ ($\delta = +0.043$) | - | 0.990 ($\delta = +0.008$) |
| | Dunn: Moderate/Minor | - | $1.049 \times 10^{-91}$ ($\delta = +0.159$) | - | 0.219 ($\delta = +0.037$) |
| | Jonckheere-Terpstra: Increasing Toxicity | $2.283 \times 10^{1}$ | 0.000 ($r = +0.091$) | 1.644 | 0.050 ($r = +0.010$) |
| | Jonckheere-Terpstra: Decreasing Toxicity | $-2.283 \times 10^{1}$ | 1.000 ($r = -0.091$) | $-1.644$ | 0.950 ($r = -0.010$) |
| **HSA** | Kruskal-Wallis | $3.531 \times 10^{2}$ | $2.157 \times 10^{-77}$ ($\eta^2 = +0.006$) | $5.199 \times 10^{2}$ | $1.285 \times 10^{-113}$ ($\eta^2 = +0.018$) |
| | Dunn: Major/Minor | - | $2.812 \times 10^{-50}$ ($\delta = +0.115$) | - | $2.128 \times 10^{-2}$ ($\delta = +0.062$) |
| | Dunn: Major/Moderate | - | $4.600 \times 10^{-47}$ ($\delta = +0.074$) | - | $4.679 \times 10^{-94}$ ($\delta = -0.176$) |
| | Dunn: Moderate/Minor | - | $3.506 \times 10^{-6}$ ($\delta = +0.038$) | - | $7.685 \times 10^{-30}$ ($\delta = +0.232$) |
| | Jonckheere-Terpstra: Increasing Toxicity | $1.874 \times 10^{1}$ | 0.000 ($r = +0.075$) | $-1.511 \times 10^{1}$ | 1.000 ($r = -0.089$) |
| | Jonckheere-Terpstra: Decreasing Toxicity | $-1.874 \times 10^{1}$ | 1.000 ($r = -0.075$) | $1.511 \times 10^{1}$ | 0.000 ($r = +0.089$) |
| **ZIP** | Kruskal-Wallis | $9.977 \times 10^{2}$ | $2.278 \times 10^{-217}$ ($\eta^2 = +0.016$) | 1.361 | 0.506 ($\eta^2 = +0.000$) |
| | Dunn: Major/Minor | - | $1.156 \times 10^{-217}$ ($\delta = +0.244$) | - | 0.900 ($\delta = -0.022$) |
| | Dunn: Major/Moderate | - | $1.592 \times 10^{-19}$ ($\delta = +0.046$) | - | 1.000 ($\delta = -0.007$) |
| | Dunn: Moderate/Minor | - | $1.688 \times 10^{-123}$ ($\delta = +0.186$) | - | 1.000 ($\delta = -0.015$) |
| | Jonckheere-Terpstra: Increasing Toxicity | $2.558 \times 10^{1}$ | 0.000 ($r = +0.102$) | $-1.090$ | 0.862 ($r = -0.006$) |
| | Jonckheere-Terpstra: Decreasing Toxicity | $-2.558 \times 10^{1}$ | 1.000 ($r = -0.102$) | 1.090 | 0.138 ($r = +0.006$) |
| **Loewe** | Kruskal-Wallis | 5.967 | 0.051 ($\eta^2 = +0.000$) | $7.641 \times 10^{1}$ | $2.554 \times 10^{-17}$ ($\eta^2 = +0.003$) |
| | Dunn: Major/Minor | - | 0.423 ($\delta = +0.012$) | - | $2.969 \times 10^{-5}$ ($\delta = +0.096$) |
| | Dunn: Major/Moderate | - | 0.076 ($\delta = +0.011$) | - | $2.570 \times 10^{-8}$ ($\delta = -0.049$) |
| | Dunn: Moderate/Minor | - | 1.000 ($\delta = -0.001$) | - | $5.530 \times 10^{-12}$ ($\delta = +0.143$) |
| | Jonckheere-Terpstra: Increasing Toxicity | 2.347 | $9.469 \times 10^{-3}$ ($r = +0.009$) | $-2.687$ | 0.996 ($r = -0.016$) |
| | Jonckheere-Terpstra: Decreasing Toxicity | $-2.347$ | 0.991 ($r = -0.009$) | 2.687 | $3.609 \times 10^{-3}$ ($r = +0.016$) |
| **S_max** | Kruskal-Wallis | $6.800 \times 10^{2}$ | $2.242 \times 10^{-148}$ ($\eta^2 = +0.011$) | $3.345 \times 10^{2}$ | $2.293 \times 10^{-73}$ ($\eta^2 = +0.012$) |
| | Dunn: Major/Minor | - | $1.692 \times 10^{-106}$ ($\delta = +0.167$) | - | $2.217 \times 10^{-8}$ ($\delta = +0.135$) |
| | Dunn: Major/Moderate | - | $8.776 \times 10^{-78}$ ($\delta = +0.097$) | - | $5.244 \times 10^{-48}$ ($\delta = -0.125$) |
| | Dunn: Moderate/Minor | - | $9.090 \times 10^{-18}$ ($\delta = +0.072$) | - | $1.143 \times 10^{-33}$ ($\delta = +0.246$) |
| | Jonckheere-Terpstra: Increasing Toxicity | $2.596 \times 10^{1}$ | 0.000 ($r = +0.104$) | $-9.133$ | 1.000 ($r = -0.054$) |
| | Jonckheere-Terpstra: Decreasing Toxicity | $-2.596 \times 10^{1}$ | 1.000 ($r = -0.104$) | 9.133 | 0.000 ($r = +0.054$) |
| **S_mean** | Kruskal-Wallis | $1.023 \times 10^{3}$ | $6.713 \times 10^{-223}$ ($\eta^2 = +0.016$) | $6.024 \times 10^{2}$ | $1.523 \times 10^{-131}$ ($\eta^2 = +0.021$) |
| | Dunn: Major/Minor | - | $1.535 \times 10^{-146}$ ($\delta = +0.195$) | - | 0.318 ($\delta = +0.048$) |
| | Dunn: Major/Moderate | - | $3.807 \times 10^{-130}$ ($\delta = +0.126$) | - | $7.979 \times 10^{-114}$ ($\delta = -0.194$) |
| | Dunn: Moderate/Minor | - | $4.827 \times 10^{-18}$ ($\delta = +0.076$) | - | $1.419 \times 10^{-28}$ ($\delta = +0.225$) |
| | Jonckheere-Terpstra: Increasing Toxicity | $3.206 \times 10^{1}$ | 0.000 ($r = +0.128$) | $-1.724 \times 10^{1}$ | 1.000 ($r = -0.101$) |
| | Jonckheere-Terpstra: Decreasing Toxicity | $-3.206 \times 10^{1}$ | 1.000 ($r = -0.128$) | $1.724 \times 10^{1}$ | 0.000 ($r = +0.101$) |
| **S_sum** | Kruskal-Wallis | $7.824 \times 10^{2}$ | $1.247 \times 10^{-170}$ ($\eta^2 = +0.012$) | $9.891 \times 10^{1}$ | $3.332 \times 10^{-22}$ ($\eta^2 = +0.003$) |
| | Dunn: Major/Minor | - | $1.198 \times 10^{-171}$ ($\delta = +0.214$) | - | $7.149 \times 10^{-6}$ ($\delta = +0.109$) |
| | Dunn: Major/Moderate | - | $1.437 \times 10^{-11}$ ($\delta = +0.035$) | - | $3.456 \times 10^{-22}$ ($\delta = +0.083$) |
| | Dunn: Moderate/Minor | - | $1.230 \times 10^{-104}$ ($\delta = +0.177$) | - | 1.000 ($\delta = +0.016$) |
| | Jonckheere-Terpstra: Increasing Toxicity | $2.208 \times 10^{1}$ | 0.000 ($r = +0.088$) | 9.640 | 0.000 ($r = +0.057$) |
| | Jonckheere-Terpstra: Decreasing Toxicity | $-2.208 \times 10^{1}$ | 1.000 ($r = -0.088$) | $-9.640$ | 1.000 ($r = -0.057$) |

**Supplementary Table 3. Drug target and pathway overlap toxicity analysis**. Results for statistical analysis of drug targets and pathway overlap correlated with toxicity categories. Each section refers to a different Jaccard Similarity metric. The first section computed the overlap between the sets of drug targets in a given drug combination, the second the sets of pathways, and the third the sets of pathways when restricted to the lowest level of Reactome. The results for the Kruskal-Wallis, Dunn with Bonferroni correction, and Jonckheere-Terpstra tests are included for both the DrugBank and DDInter datasets. The "Jonckheere-Terpstra: Increasing Toxicity" test assesses whether the distribution of the Jaccard Similarity increases when toxicity categories become more severe, while the "Jonckheere-Terpstra: Decreasing Toxicity" test evaluates if the Jaccard Similarity distributions increase when the toxicity categories decrease in severity.

| **Overlap Metric** | **Test** | **DrugBank** | | **DDInter** | |
|---|---|---|---|---|---|
| | | **Test Statistic** | ***P*-value (Effect Size)** | **Test Statistic** | ***P*-value (Effect Size)** |
| **Drug Target Jaccard Similarity** | Kruskal-Wallis | $1.533 \times 10^{2}$ | $5.254 \times 10^{-34}$ ($\eta^2 = +0.002$) | $1.565 \times 10^{2}$ | $1.053 \times 10^{-34}$ ($\eta^2 = +0.005$) |
| | Dunn: Major/Minor | - | $9.492 \times 10^{-31}$ ($\delta = +0.036$) | - | $1.352 \times 10^{-25}$ ($\delta = +0.110$) |
| | Dunn: Major/Moderate | - | $2.636 \times 10^{-11}$ ($\delta = +0.014$) | - | $9.080 \times 10^{-22}$ ($\delta = +0.039$) |
| | Dunn: Moderate/Minor | - | $1.684 \times 10^{-10}$ ($\delta = +0.022$) | - | $5.719 \times 10^{-12}$ ($\delta = +0.067$) |
| | Jonckheere-Terpstra: Increasing Toxicity | 4.782 | $8.687 \times 10^{-7}$ ($r = +0.019$) | 5.679 | $6.787 \times 10^{-9}$ ($r = +0.033$) |
| | Jonckheere-Terpstra: Decreasing Toxicity | $-4.782$ | 1.000 ($r = -0.019$) | $-5.679$ | 1.000 ($r = -0.033$) |
| **Lowest Reactome Pathways Jaccard Similarity** | Kruskal-Wallis | $1.085 \times 10^{3}$ | $2.790 \times 10^{-236}$ ($\eta^2 = +0.017$) | $1.584 \times 10^{3}$ | 0.000 ($\eta^2 = +0.055$) |
| | Dunn: Major/Minor | - | $9.492 \times 10^{-31}$ ($\delta = +0.193$) | - | $1.352 \times 10^{-25}$ ($\delta = +0.081$) |
| | Dunn: Major/Moderate | - | $2.636 \times 10^{-11}$ ($\delta = +0.097$) | - | $9.080 \times 10^{-22}$ ($\delta = +0.287$) |
| | Dunn: Moderate/Minor | - | $1.684 \times 10^{-10}$ ($\delta = +0.086$) | - | $5.719 \times 10^{-12}$ ($\delta = -0.266$) |
| | Jonckheere-Terpstra: Increasing Toxicity | $2.789 \times 10^{1}$ | 0.000 ($r = +0.111$) | $2.710 \times 10^{1}$ | 0.000 ($r = +0.159$) |
| | Jonckheere-Terpstra: Decreasing Toxicity | $-2.789 \times 10^{1}$ | 1.000 ($r = -0.111$) | $-2.710 \times 10^{1}$ | 1.000 ($r = -0.159$) |
| **All Reactome Pathways Jaccard Similarity** | Kruskal-Wallis | $1.096 \times 10^{3}$ | $8.281 \times 10^{-239}$ ($\eta^2 = +0.017$) | $7.679 \times 10^{2}$ | $1.810 \times 10^{-167}$ ($\eta^2 = +0.026$) |
| | Dunn: Major/Minor | - | $9.492 \times 10^{-31}$ ($\delta = +0.227$) | - | $1.352 \times 10^{-25}$ ($\delta = +0.049$) |
| | Dunn: Major/Moderate | - | $2.636 \times 10^{-11}$ ($\delta = +0.109$) | - | $9.080 \times 10^{-22}$ ($\delta = +0.224$) |
| | Dunn: Moderate/Minor | - | $1.684 \times 10^{-10}$ ($\delta = +0.112$) | - | $5.719 \times 10^{-12}$ ($\delta = -0.226$) |
| | Jonckheere-Terpstra: Increasing Toxicity | $3.224 \times 10^{1}$ | 0.000 ($r = +0.129$) | $2.079 \times 10^{1}$ | 0.000 ($r = +0.122$) |
| | Jonckheere-Terpstra: Decreasing Toxicity | $-3.224 \times 10^{1}$ | 1.000 ($r = -0.129$) | $-2.079 \times 10^{1}$ | 1.000 ($r = -0.122$) |

**Supplementary Table 4. Correlation analysis between drug target and pathway Jaccard Similarity and synergy scores.** Results for statistical analysis of drug targets and pathway overlap correlated with synergy scores. Each section refers to a different Jaccard Similarity metric. The first section computed the overlap between the sets of drug targets in a given drug combination, the second the sets of pathways, and the third the sets of pathways when restricted to the lowest level of Reactome. The results for the Kruskal-Wallis, Dunn with Bonferroni correction, and Jonckheere-Terpstra tests are included for both the DrugBank and DDInter datasets. The Jonckheere-Terpstra Increasing Toxicity test assesses whether the distribution of the Jaccard Similarity increases when toxicity categories become more severe, while the Jonckheere-Terpstra Decreasing Toxicity test evaluates if the Jaccard Similarity distributions increase when the toxicity categories decrease in severity.

| **Drug Target Jaccard Similarity** | | | |
|---|---|---|---|
| **Synergy Score** | **Pearson** | **Spearman** | $\boldsymbol{R}^2$ |
| Bliss | $-1.7625 \times 10^{-2}$ | $-4.0920 \times 10^{-3}$ | $3.1063 \times 10^{-4}$ |
| HSA | $-7.8355 \times 10^{-3}$ | $-2.6949 \times 10^{-2}$ | $6.1396 \times 10^{-5}$ |
| Loewe | $6.3471 \times 10^{-2}$ | $7.1984 \times 10^{-2}$ | $4.0285 \times 10^{-3}$ |
| ZIP | $-1.9607 \times 10^{-2}$ | $-6.4934 \times 10^{-3}$ | $3.8443 \times 10^{-4}$ |
| S_max | $2.0593 \times 10^{-2}$ | $-8.4737 \times 10^{-3}$ | $4.2407 \times 10^{-4}$ |
| S_mean | $-7.8945 \times 10^{-3}$ | $-4.5837 \times 10^{-2}$ | $6.2323 \times 10^{-5}$ |
| S_sum | $2.8955 \times 10^{-3}$ | $1.2554 \times 10^{-2}$ | $8.3836 \times 10^{-6}$ |
| **All Reactome Pathways Jaccard Similarity** | | | |
| **Synergy Score** | **Pearson** | **Spearman** | $\boldsymbol{R}^2$ |
| Bliss | $-1.4575 \times 10^{-3}$ | $3.4744 \times 10^{-2}$ | $2.1242 \times 10^{-6}$ |
| HSA | $-5.6955 \times 10^{-3}$ | $-2.7964 \times 10^{-2}$ | $3.2439 \times 10^{-5}$ |
| Loewe | $6.8593 \times 10^{-2}$ | $2.9483 \times 10^{-2}$ | $4.7050 \times 10^{-3}$ |
| ZIP | $-2.9755 \times 10^{-4}$ | $3.7445 \times 10^{-2}$ | $8.8535 \times 10^{-8}$ |
| S_max | $2.2987 \times 10^{-2}$ | $1.6085 \times 10^{-2}$ | $5.2840 \times 10^{-4}$ |
| S_mean | $3.2041 \times 10^{-3}$ | $-1.6027 \times 10^{-2}$ | $1.0266 \times 10^{-5}$ |
| S_sum | $2.5985 \times 10^{-2}$ | $8.2550 \times 10^{-2}$ | $6.7524 \times 10^{-4}$ |
| **Lowest Reactome Pathway Jaccard Similarity** | | | |
| **Synergy Score** | **Pearson** | **Spearman** | $\boldsymbol{R}^2$ |
| Bliss | $-6.3913 \times 10^{-3}$ | $2.8560 \times 10^{-2}$ | $4.0849 \times 10^{-5}$ |
| HSA | $-3.1004 \times 10^{-3}$ | $-4.2875 \times 10^{-2}$ | $9.6123 \times 10^{-6}$ |
| Loewe | $7.5989 \times 10^{-2}$ | $5.7598 \times 10^{-2}$ | $5.7744 \times 10^{-3}$ |
| ZIP | $-6.0386 \times 10^{-3}$ | $2.6652 \times 10^{-2}$ | $3.6465 \times 10^{-5}$ |
| S_max | $1.5437 \times 10^{-2}$ | $-1.1151 \times 10^{-2}$ | $2.3831 \times 10^{-4}$ |
| S_mean | $-9.8367 \times 10^{-5}$ | $-5.2749 \times 10^{-2}$ | $9.6761 \times 10^{-9}$ |
| S_sum | $7.9356 \times 10^{-3}$ | $5.8192 \times 10^{-2}$ | $6.2973 \times 10^{-5}$ |

**Supplementary Table 5. Toxicity scoring metrics compared to toxicity categories.** Table containing the results from statistical assessment of whether common principles used in toxicity scores trend with known DDI categories. The first section tests the similarity of drug structure in a combination by calculating the Tanimoto Similarity of the Morgan Fingerprint representations of both drugs in a combination. The second section examines whether closer average target distance in a drug combination is associated with toxicity levels. Finally, the last section calculates the overlap (Jaccard Similarity) of each drug target's neighborhood within two hops of the protein target on the STRING PPIN. The results for the Kruskal-Wallis, Dunn with Bonferroni correction, and Jonckheere-Terpstra tests are included for both the DrugBank and DDInter datasets. The "Jonckheere-Terpstra: Increasing Toxicity" assesses whether the distribution of the toxicity scoring metric increases when toxicity categories become more severe, while the "Jonckheere-Terpstra: Decreasing Toxicity" test evaluates if the toxicity scoring metric distributions increase when the toxicity categories decrease in severity.

| **Toxicity Metric** | **Test** | **DrugBank** | | **DDInter** | |
|---|---|---|---|---|---|
| | | **Test Statistic** | ***P*-value (Effect Size)** | **Test Statistic** | ***P*-value (Effect Size)** |
| **Average Target Distance** | Kruskal-Wallis | $6.672 \times 10^3$ | 0.000 ($\eta^2 = +0.106$) | $4.619 \times 10^2$ | $5.086 \times 10^{-101}$ ($\eta^2 = +0.016$) |
| | Dunn: Major/Minor | - | 0.000 ($\delta = -0.559$) | - | $8.717 \times 10^{-59}$ ($\delta = +0.312$) |
| | Dunn: Major/Moderate | - | 0.000 ($\delta = -0.264$) | - | $6.904 \times 10^{-76}$ ($\delta = +0.157$) |
| | Dunn: Moderate/Minor | - | $1.621 \times 10^{-274}$ ($\delta = -0.275$) | - | $3.232 \times 10^{-20}$ ($\delta = +0.197$) |
| | Jonckheere-Terpstra: Increasing Toxicity | $-7.878 \times 10^1$ | 1.000 ($r = -0.315$) | $2.125 \times 10^1$ | 0.000 ($r = +0.125$) |
| | Jonckheere-Terpstra: Decreasing Toxicity | $7.878 \times 10^1$ | 0.000 ($r = +0.315$) | $-2.125 \times 10^1$ | 1.000 ($r = -0.125$) |
| **Morgan Fingerprint Tanimoto Similarity** | Kruskal-Wallis | $1.084 \times 10^3$ | $3.378 \times 10^{-236}$ ($\eta^2 = +0.017$) | $1.344 \times 10^2$ | $6.481 \times 10^{-30}$ ($\eta^2 = +0.005$) |
| | Dunn: Major/Minor | - | $3.722 \times 10^{-216}$ ($\delta = +0.246$) | - | $1.516 \times 10^{-3}$ ($\delta = -0.052$) |
| | Dunn: Major/Moderate | - | 0.053 ($\delta = -0.014$) | - | $1.766 \times 10^{-20}$ ($\delta = +0.079$) |
| | Dunn: Moderate/Minor | - | $1.448 \times 10^{-208}$ ($\delta = +0.240$) | - | $1.075 \times 10^{-13}$ ($\delta = -0.160$) |
| | Jonckheere-Terpstra: Increasing Toxicity | $1.796 \times 10^1$ | 0.000 ($r = +0.072$) | 5.839 | $2.622 \times 10^{-9}$ ($r = +0.034$) |
| | Jonckheere-Terpstra: Decreasing Toxicity | $-1.796 \times 10^1$ | 1.000 ($r = -0.072$) | $-5.839$ | 1.000 ($r = -0.034$) |
| **2-Hop Neighboring Proteins Jaccard** | Kruskal-Wallis | $6.153 \times 10^3$ | 0.000 ($\eta^2 = +0.098$) | $1.019 \times 10^2$ | $7.618 \times 10^{-23}$ ($\eta^2 = +0.004$) |
| | Dunn: Major/Minor | - | 0.000 ($\delta = +0.495$) | - | 0.830 ($\delta = -0.037$) |
| | Dunn: Major/Moderate | - | 0.000 ($\delta = +0.303$) | - | $1.814 \times 10^{-22}$ ($\delta = -0.084$) |
| | Dunn: Moderate/Minor | - | $1.584 \times 10^{-99}$ ($\delta = +0.147$) | - | $8.745 \times 10^{-3}$ ($\delta = +0.064$) |
| | Jonckheere-Terpstra: Increasing Toxicity | $7.764 \times 10^1$ | 0.000 ($r = +0.310$) | $-8.154$ | 1.000 ($r = -0.048$) |
| | Jonckheere-Terpstra: Decreasing Toxicity | $-7.764 \times 10^1$ | 1.000 ($r = -0.310$) | 8.154 | $2.220 \times 10^{-16}$ ($r = +0.048$) |

# References


1. Craig Knox, Michael Wilson, Christie M. Klinger, et al. Drugbank 6.0: the drugbank knowledgebase for 2024. *Nucleic Acids Research*, 52(D1):D1265–D1275, Jan 2024. doi: 10.1093/nar/gkad976.

2. Guoli Xiong, Zhijiang Yang, Jiacai Yi, Ningning Wang, Lei Wang, Huimin Zhu, Chengkun Wu, Aiping Lu, Xiang Chen, Shao Liu, Tingjun Hou, and Dongsheng Cao. Ddinter: an online drug-drug interaction database towards improving clinical decision-making and patient safety. *Nucleic Acids Research*, 50(D1):D1200–D1207, Jan 2022. doi: 10.1093/nar/gkab880. PMID: 34634800; PMCID: PMC8728114.

3. Bulat Zagidullin, Jehad Aldahdooh, Shuyu Zheng, Wenyu Wang, Yinyin Wang, Joseph Saad, Alina Malyutina, Mohieddin Jafari, Ziaurrehman Tanoli, Alberto Pessia, and Jing Tang. Drugcomb: an integrative cancer drug combination data portal. *Nucleic Acids Research*, 47(W1):W43–W51, 05 2019. ISSN 0305-1048. doi: 10.1093/nar/gkz337. URL `https://doi.org/10.1093/nar/gkz337`.

4. Susan L. Holbeck, Richard Camalier, James A. Crowell, Jeevan Prasaad Govindharajulu, Melinda Hollingshead, Lawrence W. Anderson, Eric Polley, Larry Rubinstein, Apurva Srivastava, Deborah Wilsker, Jerry M. Collins, and James H. Doroshow. The national cancer institute almanac: A comprehensive screening resource for the detection of anticancer drug pairs with enhanced therapeutic activity. *Cancer Research*, 77(13):3564–3576, 2017.

5. Jennifer O'Neil, Yair Benita, Igor Feldman, Melissa Chenard, Brian Roberts, Yaping Liu, Jing Li, Astrid Kral, Serguei Lejnine, Andrey Loboda, William Arthur, Razvan Cristescu, Brian B. Haines, Christopher Winter, Theresa Zhang, Andrew Bloecher, and Stuart D. Shumway. An unbiased oncology compound screen to identify novel combination strategies. *Molecular Cancer Therapeutics*, 15(6):1155–1162, 2016.

6. Marco P Licciardello, Anna Ringler, Patrick Markt, Freya Klepsch, Charles-Hugues Lardeau, Sara Sdelci, Erika Schirghuber, André C Müller, Michael Caldera, Anja Wagner, Rebecca Herzog, Thomas Penz, Michael Schuster, Bernd Boidol, Gerhard Dürnberger, Yasin Folkvaljon, Pär Stattin, Vladimir Ivanov, Jacques Colinge, Christoph Bock, Klaus Kratochwill, Jörg Menche, Keiryn L Bennett, and Stefan Kubicek. A combinatorial screen of the cloud uncovers a synergy targeting the androgen receptor. *Nature Chemical Biology*, 13(7):771–778, Jul 2017. doi: 10.1038/nchembio.2382. Epub 2017 May 22; PMID: 28530711.

7. Giovanni C Forcina, Megan Conlon, Alex Wells, Jennifer Yinuo Cao, and Scott J Dixon. Systematic quantification of population cell death kinetics in mammalian cells. *Cell Systems*, 4(6): 600–610.e6, Jun 2017. doi: 10.1016/j.cels.2017.05.002. Epub 2017 Jun 7; PMID: 28601558; PMCID: PMC5509363.

8. A. Ianevski, K. A. Giri, and T. Aittokallio. SynergyFinder 3.0: an interactive analysis and consensus interpretation of multi-drug synergies across multiple samples. *Nucleic Acids Research*, page gkac382, 2022. doi: 10.1093/nar/gkac382.

9. Alina Malyutina, Muntasir Mamun Majumder, Wenyu Wang, Alberto Pessia, Caroline A. Heckman, and Jing Tang. Drug combination sensitivity scoring facilitates the discovery of synergistic and efficacious drug combinations in cancer. *PLOS Computational Biology*, 15(5):1–19, 05 2019. doi: 10.1371/journal.pcbi.1006752. URL `https://doi.org/10.1371/journal.pcbi.1006752`.
